# Rational Design of Ruddlesden–Popper Perovskite Ferrites as Air Electrode for Highly Active and Durable Reversible Protonic Ceramic Cells

**DOI:** 10.1007/s40820-024-01397-2

**Published:** 2024-04-22

**Authors:** Na Yu, Idris Temitope Bello, Xi Chen, Tong Liu, Zheng Li, Yufei Song, Meng Ni

**Affiliations:** 1 Department of Building and Real Estate, Research Institute for Sustainable Urban Development (RISUD) and Research Institute for Smart Energy (RISE), The Hong Kong Polytechnic University, Hung Hom, Kowloon, Hong Kong, People’s Republic of China; 2 The Hong Kong Polytechnic University Shenzhen Research Institute, Shenzhen, 518057 Guangdong People’s Republic of China; 3 Department of Mechanical and Aerospace Engineering, The Hong Kong University of Science and Technology, Clear Water Bay, Hong Kong, People’s Republic of China

**Keywords:** Reversible protonic ceramic cells, Air electrode, Ruddlesden–Popper perovskite, Hydration, Oxygen reduction reaction

## Abstract

Reversible protonic ceramic cells (RePCCs) hold promise for efficient energy storage, but their practicality is hindered by a lack of high-performance air electrode materials. Ruddlesden–Popper perovskite Sr_3_Fe_2_O_7−δ_ (SF) exhibits superior proton uptake and rapid ionic conduction, boosting activity. However, excessive proton uptake during RePCC operation degrades SF’s crystal structure, impacting durability. This study introduces a novel A/B-sites co-substitution strategy for modifying air electrodes, incorporating Sr-deficiency and Nb-substitution to create Sr_2.8_Fe_1.8_Nb_0.2_O_7−δ_ (D-SFN). Nb stabilizes SF's crystal, curbing excessive phase formation, and Sr-deficiency boosts oxygen vacancy concentration, optimizing oxygen transport. The D-SFN electrode demonstrates outstanding activity and durability, achieving a peak power density of 596 mW cm^−2^ in fuel cell mode and a current density of − 1.19 A cm^−2^ in electrolysis mode at 1.3 V, 650 °C, with excellent cycling durability. This approach holds the potential for advancing robust and efficient air electrodes in RePCCs for renewable energy storage.

**Supplementary Information:**

The online version contains supplementary material available at 10.1007/s40820-024-01397-2.

## Highlights

- A novel A/B-sites co-substitution strategy was introduced to enhance the performance and durability of Ruddlesden–Popper perovskite Sr_3_Fe_2_O_7−δ_ (SF)-based air electrodes for reversible protonic ceramic cells (RePCCs).
- Simultaneous Sr-deficiency and Nb-substitution in SF result in Sr_2.8_Fe_1.8_Nb_0.2_O_7−δ_ (D-SFN), offering improved structural stability under RePCC conditions by suppressing the formation of Sr_3_Fe_2_(OH)_12_ phase.
- The introduction of Sr-deficiency enhances oxygen vacancy concentration in D-SFN, promoting efficient oxygen transport within the material and contributing to excellent activity in RePCCs.

**Supplementary Information:**

The online version contains supplementary material available at 10.1007/s40820-024-01397-2.

## Graphical abstract

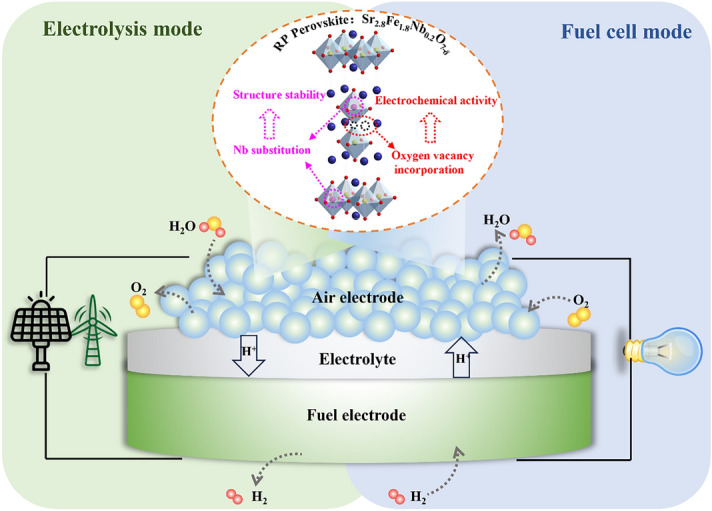

## Introduction

The pressing need to address environmental concerns has intensified the quest for renewable energy sources and their corresponding energy storage solutions [1, 2]. Traditional batteries like lead-acid and lithium-ion batteries have been considered but are plagued by issues like toxicity (lead-acid batteries), limited energy storage capacity, high cost, and safety concerns (Li-ion batteries) that make them less ideal for long-term energy storage applications in renewable energy capture [3, 4]. Alternative energy conversion devices, such as proton exchange membrane fuel cells (PEMFCs) designed for low-temperature operation (< 100 °C) and solid oxide cells (SOCs) suited for high-temperature operation (700–1000 °C), have garnered attention [5, 6]. Nonetheless, PEMFCs exhibit suboptimal energy conversion efficiency at low temperatures and often rely on noble metal catalysts. In contrast, SOCs have higher energy conversion efficiencies and typically employ non-noble metal catalysts. However, extended high-temperature operation above 700 °C accelerates component aging, necessitating stringent compatibility requirements and posing challenges to long-term operation [7].

**Fig. 1:**
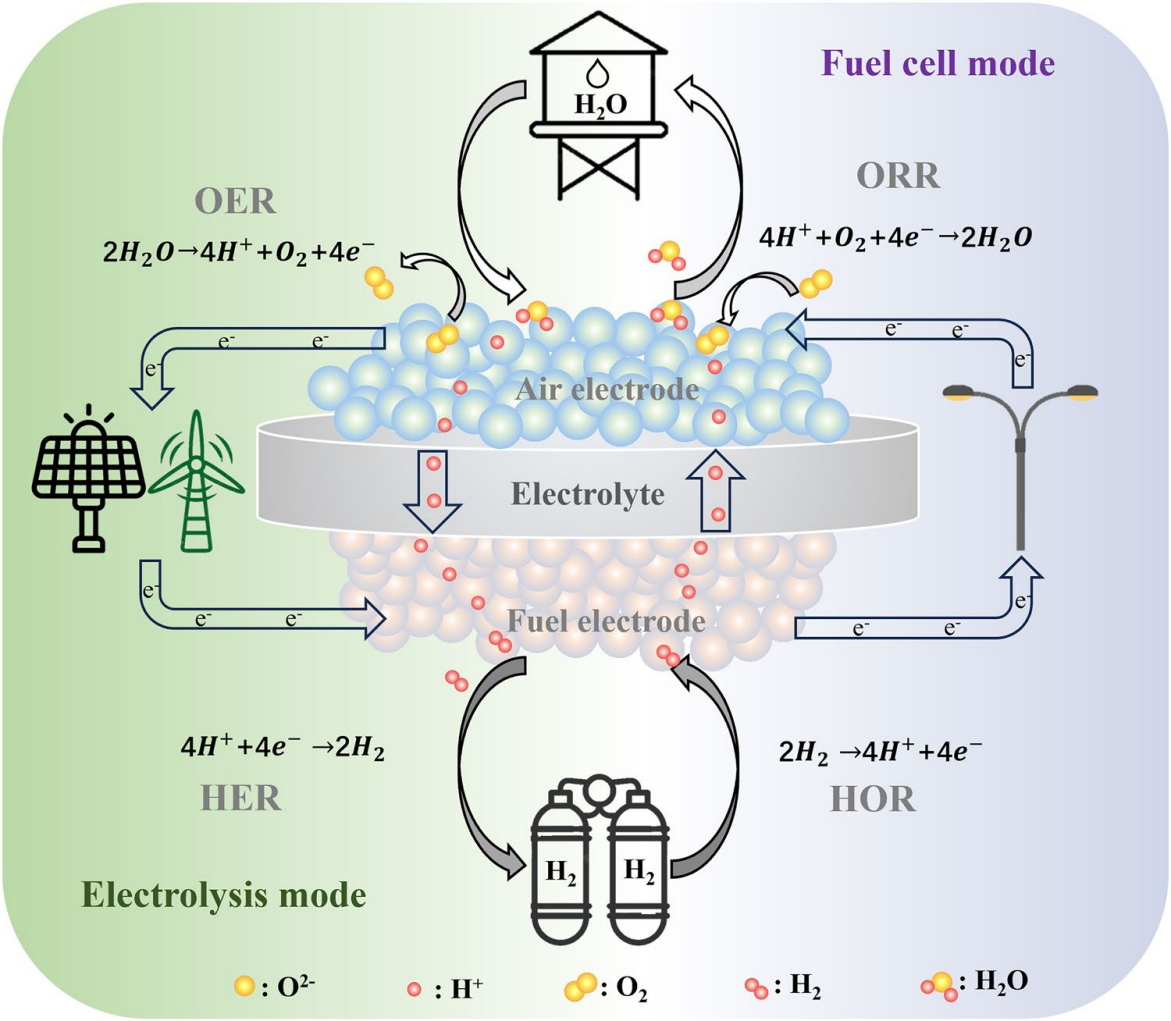
Working principle of RePCC combined with renewable energy

Researchers have innovatively introduced SOCs with proton conductors as electrolyte materials, termed reversible protonic ceramic cells (RePCCs). By utilizing proton conductors with significantly lower activation energies for ion transport than traditional oxygen-ion conductors, RePCCs can operate efficiently at intermediate temperatures of 350–600 °C [8, 9]. This moderated temperature range balances high performance and stability. RePCCs allow reversible switching between protonic ceramic fuel cell (PCFC) mode for electricity generation and protonic ceramic electrolysis cell (PCEC) mode for electrolytic hydrogen production (Fig. 1). In PCEC mode, RePCCs can store renewable electricity as chemical energy by electrolyzing water to hydrogen. Conversely, in PCFC mode, the chemical energy in hydrogen is converted to electrical power. Notably, RePCCs generate pure, dry hydrogen in PCEC mode without external gas purification, substantially improving system efficiency [10]. The operational reversibility enables optimal energy storage during renewable electricity oversupply and electricity generation to meet demand. However, the reduced operating temperature presents challenges for achieving sufficient catalytic activity in air electrode materials during the pivotal oxygen reduction reaction (ORR) and water oxidation reaction (WOR) involving H^+^/O^2-^ transport [11, 12]. The sluggish kinetics of these electrochemical reactions at intermediate temperatures likely relate to obstructed oxygen surface exchange processes and proton conduction issues [13, 14]. Thus, the development of suitable air electrode materials plays a pivotal role in enhancing RePCC performance.

Among various electrode materials, Ruddlesden–Popper (RP) perovskite oxide Sr_3_Fe_2_O_7−δ_ (SF) is a promising RePCC air electrode candidate because of the uniqueness of its layered structure. Arranged by alternating a layer of rock salt (SrO) and n layers of perovskite (SrFeO_3_), this unique structure can accommodate a large number of oxygen defects, and at the same time endows SF material with anisotropic defect transport properties, which is favorable for proton defects formation and migration [15, 16]. Another advantage of SF materials as RePCC air electrodes is their excellent water storage properties (hydration) [17, 18]. This introduces proton defects, which is usually considered favorable for proton conductivity. However, the rapid hydration of SF under RePCCs condition easily leads to an excessive formation of the Sr_3_Fe_2_ (OH)_12_ (SFH) phase [16], destroying the main phase of SF, thereby potentially resulting in poor cell operational stability. Hence, the key to developing highly active and stable SF-based air electrode materials lies in stabilizing the crystal structure to achieve optimal content of the SFH phase without excessive hydration.

Doping high oxidation state elements such as Ti^4+^, Zr^4+^, and Nb^5+^ is a common strategy to improve the durability of materials [19–21]. Notably, Nb^5+^ presents a higher oxidation state and stronger Nb–O bonds, rendering it an attractive dopant for improving material stability [22, 23]. However, this strategy may adversely affect the electrochemical performance because the combination of high valence elements reduces the oxygen vacancies in the material, while oxygen vacancy is vital in oxygen ion transport and hydration reactions [24]. Meanwhile, A-site vacancy is a common strategy to increase the oxygen vacancy concentration of perovskite materials to maintain oxygen transport characteristics.

Inspired by these studies, here, we propose a simple A/B sites co-substitution strategy to design and develop a SF-based perovskite air electrode for RePCCs technology. In this work, Sr-deficiency and Nb-substitution are simultaneously introduced into SF to make up Sr_2.8_Fe_1.8_Nb_0.2_O_7–δ_ (D-SFN). The Nb-substitution in SF stabilizes the crystal structure under RePCCs condition, suppressing the excessive formation of the SFH phase, thereby ensuring the stability of the major SF structure. In addition, the incorporation of Sr-deficiency further increases the oxygen vacancy concentrations, promoting oxygen transport characteristics. As a result, D-SFN showed both enhanced electrochemical performance and durability. D-SFN-based RePCCs achieved a peak power density of 596 mW cm^−2^ at 650 °C in fuel cell mode. It also attained a current density of − 1.19 A cm^-2^ in the electrolysis mode at 1.3 V, under hydrogen and humidified air feeds. When operated reversibly between PCFC and PCEC modes, stable performance was maintained 160 h, encompassing 20 cycles. This work can insprie the design and development of RP-type perovskite air electrode materials for RePCCs, thereby accelerating the commercialization of this RePCCs.

## Experimental

### Material Fabrication

The SF, SFN, D-SFN (S_3−*y*_FN_*x*_, x = 0, 0.2; y = 0, 0.2), and BZCYYb materials were prepared by the sol–gel method [25]. For S_3−*y*_FN_*x*_, Sr(NO_3_)_2_ (AR, ≥ 99.5%), Fe(NO_3_)_3_·9H_2_O (AR, ≥ 98.5%), C_10_H_5_NbO_20_ (AR, ≥ 98%) as the chemical reagents were stoichiometrically dissolved in deionized water on a magnetic heating stirrer at 80 °C until gel formation occurred. The citric acid (AR, ≥ 99.5%) and EDTA (AR, ≥ 99.5%) were added as complexing agents, and ammonia water (25%–28%) was employed to make the solution neutral or slightly alkaline. Subsequently, the gel was heated in the oven at 180 °C for 10 h to obtain the precursor, which was then calcined at 1100 °C for 10 h to obtain the initial S_3–*y*_FN_*x*_ powders.

### Material Characterization

An X-ray diffractometer (XRD, Rigaku SmartLab 9 kW) was employed to record diffraction patterns of the powder samples within the 10°–80° ranges using a step-scan mode with 0.02° intervals. X-ray photoelectron spectroscopy (XPS, Thermo Fisher Scientific Nexsa) was utilized to investigate the elemental valence states on the S_3−*y*_FN_*x*_ surface. A field emission scanning electron microscope (SEM, Tescan MIRA) and scanning transmission electron microscopy (STEM, FEI Talos F200x) were used to observe the cross-sectional microstructure of the single cell. A high-resolution transmission electron microscope (HR-TEM, FEI Talos F200x) was employed to observe the lattice spacing of fresh powder samples, and energy-dispersive X-ray spectroscopy (EDX, FEI Talos F200x) mapping was used to obtain element distributions. Inductively coupled plasma optical emission spectroscopy (ICP-OES, Thermo Scientific iCAP 7600) was employed for precise compositional analysis. The oxygen vacancy content of S_3−*y*_FN_*x*_ samples at room temperature was determined via iodometric titration. Detailed experimental information is available in the supporting information. Thermogravimetry analysis (TGA, TGA5500) was conducted from 40 to 700 °C. The average linear thermal expansion coefficient (TEC) of the S_3−*y*_FN_*x*_ samples was measured using a dilatometer (DIL 402CL, Netzsch) in air, with a heating rate of 10 °C min^−1^. The fourier transform infrared (FT-IR, Thermo Scientific Nicolet iS5) spectra were used to examine the hydration degree of the samples, with a scanning range from 1500 to 4000 cm^−1^. Before FT-IR test, the samples were subjected to a 5-h drying process in a 100 °C oven to eliminate adsorbed water from the sample surfaces. The electrical conductivity of the S_3−*y*_FN_*x*_ was examined by the DC four-probe method. The specific experimental details can be found in the supporting information.

### Electrochemical Performance Test

Electrochemical impedance spectroscopy (EIS) test was done from 10^5^ to 10^–1^ Hz by an electrochemical workstation (Solarton 1287 + 1260), with a signal amplitude of 30 mV. *I-V-p* curve and Ni-BZCYYb|BZCYYb|S_3−*y*_FN_*x*_ single cell stability tests were measured by a digital source meter (Keithley 2440). The single cell was supplied with 50 sccm of H_2_ as fuel and 100 sccm of synthetic air as oxidant. Water vapor was introduced via a water bath heating method. Furthermore, the distribution of relaxation time (DRT) method was employed to comprehensively understand the EIS results [26, 27]. Details about DRT can be found in the supporting information.

### Computational Details

The first-principles computations were conducted within the framework of DFT using VASP [28, 29]. The exchange–correlation interaction was treated employing a generalized gradient approximation (GGA) as described by the Perdew-Burke-Ernzerhof (PBE) function [30]. The kinetic energy cutoff of the plane wave utilized to extend the Kohn–Sham electron wave function was set to 400 eV, with an iterative convergence of energy being 10^–5^ eV. All atomic positions were allowed to relax until the Hellmann–Feynman force fell below 0.01 eV Å^−1^. The Brillouin zone was sampled by a 2 × 2 × 2 k-point grid. Further specifics of the calculation are elucidated in the supporting information.

## Results and Discussion

### Analysis of Composition and Structure

**Fig. 2:**
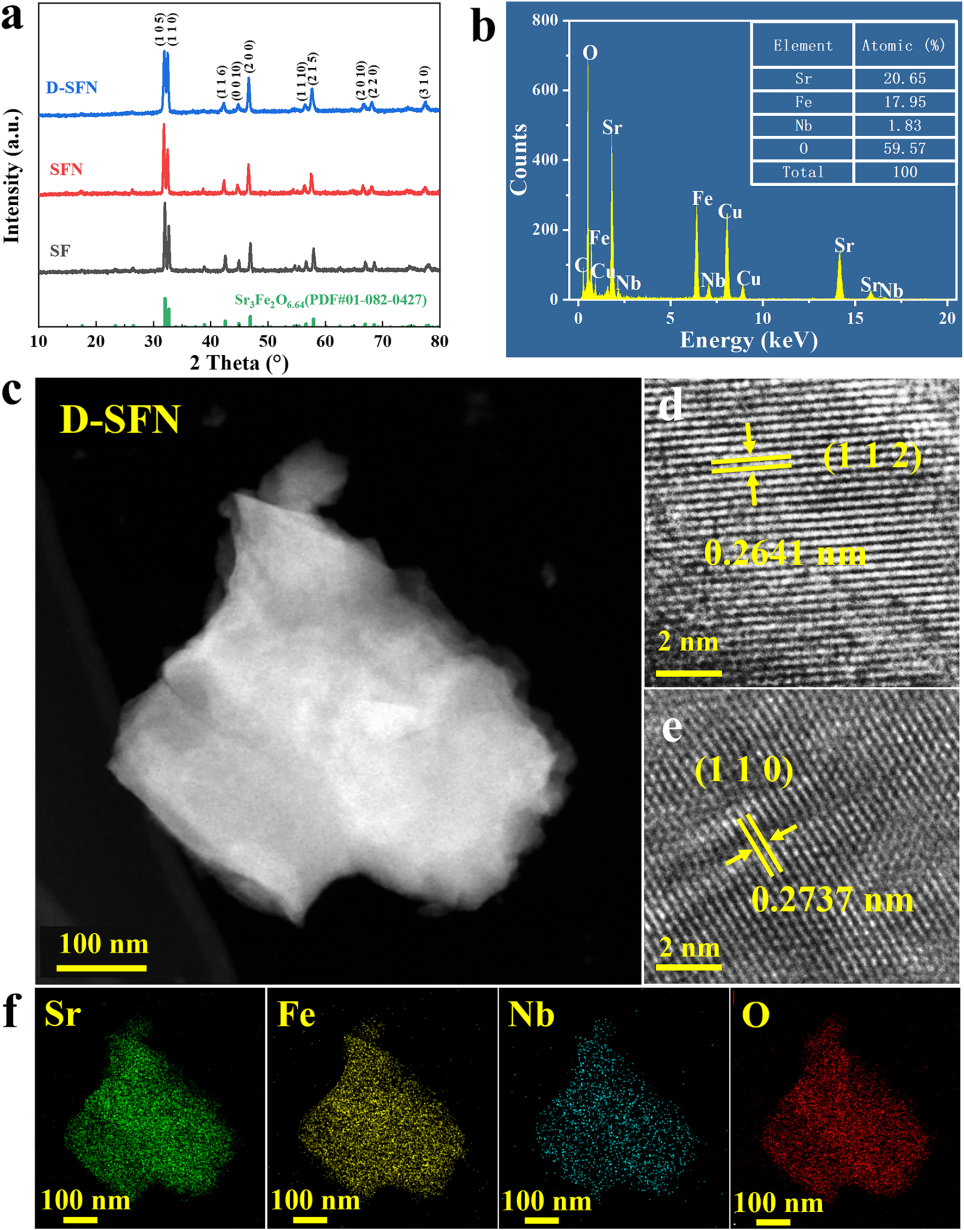
Structural characterization of S_3−*y*_FN_*x*_ compositions. **a** XRD patterns indexed to standard RP structure. **b** EDX confirming elemental composition. **c** STEM showing nanoparticle morphology. **d, e** HR-TEM verifying crystallinity and d-spacings. **f** EDX mapping demonstrating uniform Nb incorporation

To illustrate the respective effects of Nb doping and A-site cation defects on the material properties, three perovskite precursors with different compositions were designed, namely, the initial Sr_3_Fe_2_O_7-δ_ (SF) material, the Nb-doped Sr_3_Fe_1.8_Nb_0.2_O_7-δ_ (SFN), and the simultaneous Nb-doped and A-site deficient Sr_2.8_Fe_1.8_Nb_0.2_O_7-δ_ (D-SFN). The successful synthesis and crystallinity of SF, SFN, and D-SFN compositions via the sol–gel method were verified using XRD, as shown in Fig. 2a. All patterns were indexed to the standard Ruddlesden–Popper A_3_B_2_O_7-δ_ perovskite structure (PDF#01-082-0427), with no detectable impurities. This confirms the incorporation of Nb into the SF lattice for SFN and D-SFN. In perovskites, the BO_6_ octahedron structurally supports the framework, enabling A-site Sr vacancies in D-SFN without instability. Further Rietveld refinement quantified the lattice parameters (Fig. S1, Tables S1-S4). Excellent refinement fitting (*R*_P_ < 10%, *R*_WP_ < 15%) validated the reliability. The successful doping of Nb and the introduction of A-site defects are further supported by the partially expanded lattice after Nb doping (301.4 Å^3^ for SF compared to 305.5 Å^3^ for SFN) and the subsequent partially contracted unit cell of D-SFN (302.7 Å^3^). STEM revealed uniform nanoparticle morphologies for D-SFN with diameters of ~ 200–300 nm (Fig. 2c). HR-TEM verified the crystallinity through measured d-spacings of 0.2641 and 0.2737 nm, matching the (112) and (110) planes (Fig. 2d, e). EDX confirmed the expected elemental composition (Fig. 2b). Given that EDX is a semi-quantitative analysis method, the elemental composition of fresh D-SFN samples was further determined using ICP-OES. The results indicate that the proportions of Sr, Fe, and Nb in the cationic total are 50.63%, 20.04%, and 4.00%, respectively, closely aligning with the nominal values of 51.49%, 21.10%, and 3.90%, with only minor deviations observed. EDX mapping further verified the successful homogeneous substitution of Nb into the parent SF lattice (Fig. 2f).

### Analysis of Physicochemical Properties

**Fig. 3:**
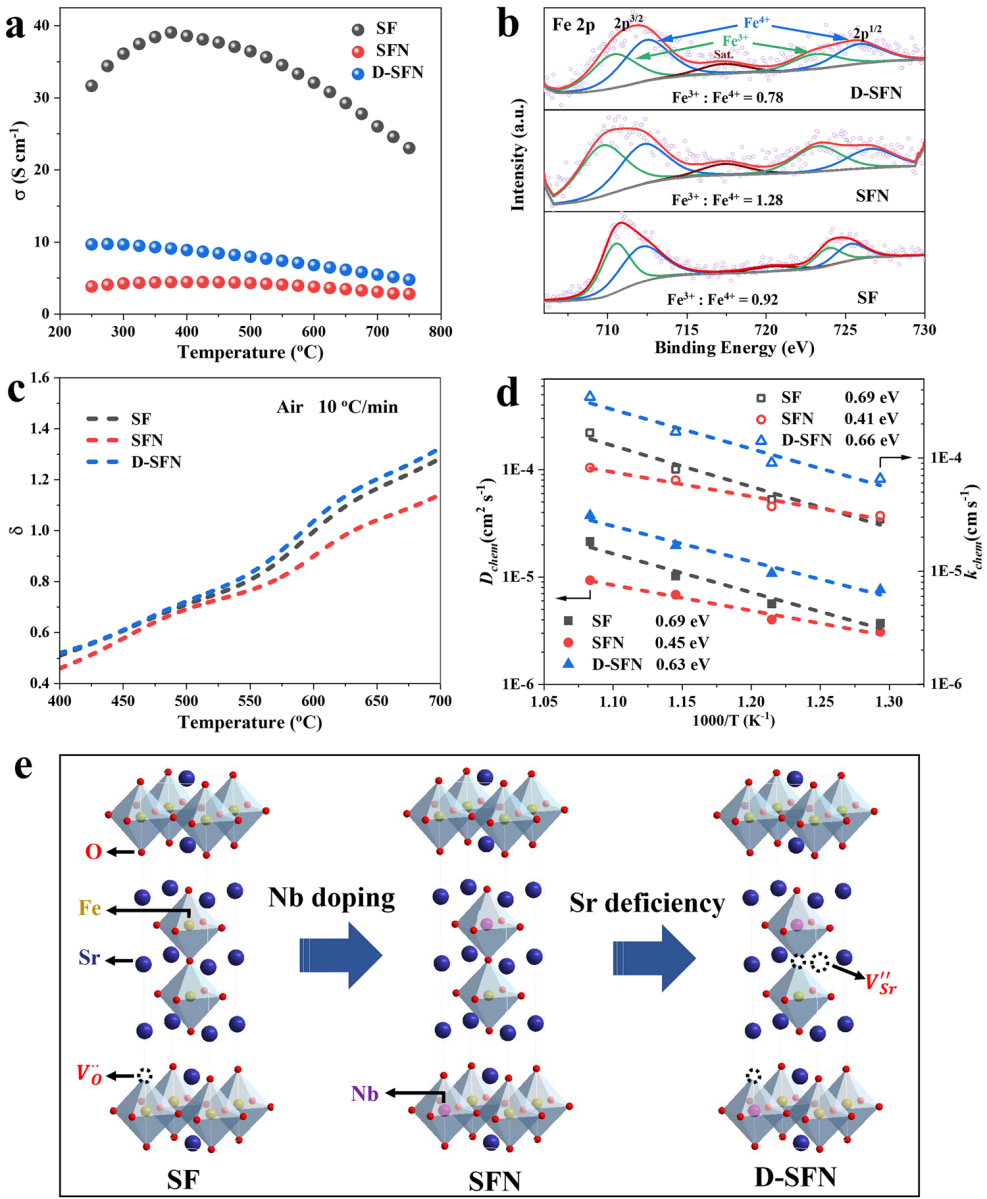
Impact of Nb doping and Sr deficiency on S_3−*y*_FN_*x*_ properties. **a** Electrical conductivity showing the semiconducting-metallic transition. **b** XPS valence state quantification of Fe 2*p*. **c** Oxygen non-stoichiometry (δ) determination via TGA and iodometric test. **d** Oxygen surface exchange coefficient (*k*_*chem*_) and oxygen bulk diffusion coefficient (*D*_*chem*_) from ECR. **e** Schematic diagram of the dual modification strategy

For the air electrode of RePCC, the excellent electronic conductivity helps to realize the electronic conducting path and provides the necessary conditions for the electrochemical reaction to proceed [31]. Herein, the electronic conductivity of S_3−*y*_FN_*x*_ materials was investigated using the DC four-probe method within a temperature range of 250–750 °C (Fig. 3a). The electrical conductivity profile of SF material exhibits remarkable temperature-dependent behavior, initially increasing with temperature likely due to thermal excitation of charge carriers, followed by a noticeable decline around 400 °C. This non-linear behavior indicates a transition from semiconducting to metallic characteristics, which is due to the lattice oxygen loss at elevated temperatures according to the following defect reaction [32]:

1 $$ 2{\text{O}}_{{\text{O}}}^{ \times } + 4h^{\cdot} \to {\text{O}}_{2} + 2V_{{\text{O}}}^{\cdot\cdot} $$

As temperature rises, the reduced Fe facilitates the escape of lattice oxygen, forming oxygen vacancy (${V}_{O}^{\cdot \cdot }$) and releasing O_2_. Concurrently, the electron holes (${h}^{\cdot }$) were consumed, causing the decreased conductivity. Incorporating Nb led to a substantial reduction in electrical conductivity compared to the pristine SF material. Over 250–750 °C, the conductivity of SFN was only 2.8–4.46 S cm^−1^, much lower than the 23.04–39.06 S cm^−1^ range exhibited by SF. This considerable decrease suggests Nb^5+^ ions inhibit electron mobility in the material [33].

However, combining Nb doping and A-site deficiency in D-SFN notably enhanced its electrical conductivity compared to SFN, doubling it to 4.79–9.73 S cm^−1^ over 250–750 °C. This indicates that introducing Sr vacancies can compensate for the reduced conductivity induced by Nb doping. To comprehend the mechanisms contributing to the enhanced conductivity in D-SFN, an exploration of the electronic structures of SFN and D-SFN was undertaken utilizing density of states (DOS) plots. After calibration, 0 eV corresponds to the Fermi level (E_f_). As depicted in Fig. S2, the conduction band near the Fermi level is predominantly shaped by Fe 3*d* orbitals, while the valence band primarily originates from O 2*p* orbitals. This underscores the vital role of Fe–O interactions in influencing the electronic conductivity of the material. The broadening of unoccupied states around the valence band in D-SFN implies an augmentation in charge transfer [34, 35]. Introducing Sr defects leads to an increase in the Fe valence states, resulting in an amplified overlap of Fe 3*d* and O 2*p* orbitals near the Fermi level. This indicates a reinforcement of Fe–O covalency, favorably impacting electron conduction in the material [36, 37]. Nonetheless, the conductivity remains inferior to that of pristine SF, highlighting the intricate interplay between doping and deficiency in influencing material properties. However, when the air electrode meets the electronic conductivity of 1 S cm^−1^, it is enough to provide the required electron transport path for electrochemical reactions [38, 39]. Therefore, the electronic conductivities of the three materials can meet the needs of high-performance air electrodes.

In addition to sufficient electronic conductivity, an ideal RePCC air electrode should also present high O^2−^/H^+^ conductivity and surface exchange performance. These properties are usually related to the B-site transition metal oxidation state, oxygen vacancy concentration, and material surface chemical properties [40]. Therefore, XPS was introduced to get information about surface chemistry and oxidation states in SF, SFN, and D-SFN. The Nb 3*d* peaks observed around 209 eV in the SFN and D-SFN survey scans (Fig. S3) confirm the successful doping of Nb into the parent SF material. Deconvoluted high-resolution XPS spectra of Fe 2*p* reveal a mixed Fe^3+^/Fe^4+^ valence state in all samples (Fig. 3b) [41, 42]. Compared with SF, the Fe valence state of SFN is significantly reduced, while the Fe valence state of D-SFN is slightly increased. Relative Fe^3+^ and Fe^4+^ percentages were adopted to estimate δ of the surface, presented in Table S5. Nb doping induces a decrease in the δ from 0.48 in SF to 0.41 in SFN, indicating Nb incorporation reduces surface oxygen vacancies. However, A-site deficiency in D-SFN marginally increases δ to 0.49 and is even slightly higher than that of SF.

It is generally accepted that the air electrode of RePCC, in a humid atmosphere, undergoes hydration reactions by the combination of oxygen vacancies on the electrode surface with steam, resulting in the generation of proton defects [43, 44], as indicated by the following equation:

2 $$ {\text{H}}_{2} {\text{O}} + {\text{O}}_{{\text{O}}}^{ \times } + V_{{\text{O}}}^{\cdot\cdot} \rightarrow 2{\text{OH}}_{{\text{O}}}^{\cdot} $$

Therefore, the surface oxygen vacancy content is generally indicative of the material's hydration performance partially. It is anticipated that D-SFN, possessing a higher concentration of oxygen vacancies due to Sr deficiency, would exhibit superior hydration performance compared to SFN. FT-IR testing was employed to ascertain the hydration characteristics of SFN and D-SFN samples. As shown in Fig. S4, both samples revealed infrared absorption peaks corresponding to ${{\text{OH}}}_{{\text{O}}}^{\cdot }$ in the range of 3200–3700 cm^−1^ after complete hydration (3% H_2_O-air for 150 h). Similar phenomena have been observed in other perovskite air electrode materials, such as BaCo_0.7_Fe_0.2_Zr_0.1_O_3−δ_ [45] and BaCo_0.7_Ce_0.24_Y_0.06_O_3−δ_ [46]. The ${{\text{OH}}}_{{\text{O}}}^{\cdot }$ absorption peak in D-SFN is more pronounced than in SFN, indicating enhanced hydration capabilities.

Considering that lattice oxygen also participates in the ORR/WOR reaction of RePCC, excellent bulk oxygen migration properties are also significant to the ORR/WOR activity of the material [47, 48]. The bulk oxygen vacancy content of the materials was determined through the iodometric method. The average oxygen vacancy contents of SF, SFN, and D-SFN are marginally higher than the surface oxygen vacancy contents, measuring 0.51, 0.46, and 0.52, respectively. Discrepancies between XPS and iodometric results could stem from variations in the valence state of the Fe element between the bulk and surface regions of the materials [49]. Compared with Fe ions in the bulk phase, Fe ions on the surface are more fully in contact with air and are more easily oxidized [50].

In addition, the lattice oxygen activity of the materials at high temperatures was investigated based on TG. Shown in Fig. S5, the pronounced mass loss observed above ~ 400 °C in all samples has been attributed to the thermal reduction of Fe and subsequent release of lattice oxygen [51, 52], which confirms the change of the conductivity in Fig. 3a.

Since RePCCs usually operate at intermediate temperatures of 400–700 °C, integrating iodometry and TGA results yielded the most representative oxygen vacancy content of the air electrode material in the operational state. The oxygen vacancy evolution of SF, SFN, and D-SFN in the corresponding operating temperature ranges was depicted in Fig. 3c. Compared to SF, SFN has a lower δ value at 400–700 °C. For example, at 550 °C, the δ value for SF is 0.81, whereas for SFN, it is only 0.77. Considering that the bond strength of the Nb–O bond is significantly higher than that of the Fe–O bond, the lattice oxygen combined with Nb is more stable than that combined with Fe [53, 54]. Therefore, after doping Nb, the overall activity of lattice oxygen in the bulk phase is weakened, O^2−^ ions are less likely to escape from the lattice when the temperature increases and the oxygen vacancy concentration decreases. D-SFN has the highest δ value of 1.32 at 700 °C, which may be related to the increased lattice oxygen activity near the Sr defects.

The thermal compatibility between the air electrode and electrolyte material is crucial for the practical application of a single cell. Mismatched TEC between electrolyte and electrode can induce residual stresses, leading to interfacial delamination [55]. The thermal expansion behavior of the materials under an air atmosphere was examined using thermal expansion tests, as shown in Fig. S6. The average TEC values for SF, SFN, and D-SFN from 100 to 1000 °C were 18.3 × 10^–6^, 16.5 × 10^–6^, and 17.1 × 10^–6^ K^−1^, respectively, significantly lower than the TEC of some typical Co-based air electrodes [56, 57]. Despite Sr defects causing unit cell contraction, partially aiding in the improvement of the TEC, the material's high-temperature expansion behavior is primarily attributed to the reduction of B-site elements and lattice oxygen loss [58, 59]. At elevated temperatures, the TEC of D-SFN is slightly higher than SFN, possibly due to the greater reduction of Fe^4+^ in D-SFN at high temperatures, as evidenced by TG and XPS results. However, it should be noted that SFN and D-SFN exhibit very close TEC within the operational temperature range (500–750 °C), highlighting the beneficial impact of Nb incorporation. Considering the relatively low average TEC of BZCYYb (9.5 × 10^–6^ K^−1^) [60], the introduction of Nb can decrease the TEC of the SF-based air electrode, enhancing the long-term thermal compatibility with BZCYYb electrolyte during operation.

To further evaluate the oxygen surface exchange and bulk diffusion properties of the electrode material in a real environment, the chemical bulk diffusion and surface exchange kinetics of the material when the oxygen partial pressure changes were tested through ECR experiments. The experimental process has been given in detail in the experimental section. Table S6 summarizes the bulk oxygen diffusion coefficient (*D*_*chem*_) and surface oxygen exchange coefficient (*k*_*chem*_) of SF, SFN, and D-SFN between 500 and 650 °C quantified by the ECR curve (Fig. S7). The Arrhenius curves of *k*_chem,_ and* D*_*chem*_ of the three materials at 500–650 °C were plotted in Fig. 3d. Compared with SF, SFN exhibits lower *D*_*chem*_ and *k*_*chem*_ values between 500 and 650 °C, indicating that niobium doping may adversely affect bulk and surface oxygen transport [61]. The introduction of A-site defects in SFN can significantly improve the oxygen transport properties of the material. Compared to SFN, the *D*_*chem*,_ and *k*_*chem*_ of D-SFN increased by 1.9 and 1.7 times respectively, at 600 °C. The oxygen vacancies introduced by Sr defects and the improved lattice oxygen activity effectively improve the bulk diffusion and surface exchange kinetics of SFN [62]. These ECR results highlight the efficacy of A-site vacancies in enhancing the oxygen transport capability of niobium-doped S_3−*y*_FN_*x*_ compositions.

Based on the above discussion, the mechanism by which niobium incorporation and strontium defects manipulate material properties can be represented by the schematic diagram in Fig. 3e and the following Kröger–Vink notation:

Nb doping:

3 $$ {\text{Nb}}_{2} {\text{O}}_{5} + V_{{\text{O}}}^{\cdot\cdot} + 2{\text{Fe}}_{{{\text{Fe}}}}^{ \times } \to 2{\text{Nb}}_{{{\text{Fe}}}}^{\cdot} + {\text{O}}_{{\text{O}}}^{ \times } + 2{\text{O}}_{2} $$

A-site deficiency:

4 $$ {\text{Sr}}_{{{\text{Sr}}}}^{ \times } + {\text{O}}_{{\text{O}}}^{ \times } \to V^{\prime\prime}_{{{\text{Sr}}}} + V_{{\text{O}}}^{\cdot\cdot} + {\text{SrO}} $$

5 $$ 2{\text{Fe}}_{{{\text{Fe}}}}^{ \times } + {\text{Sr}}_{{{\text{Sr}}}}^{ \times } + \frac{1}{2}{\text{O}}_{2} \to V^{\prime\prime}_{{{\text{Sr}}}} + 2{\text{Fe}}_{{{\text{Fe}}}}^{\cdot} + {\text{SrO}} $$

Nb^5+^ substitution on Fe sites consumes oxygen vacancies (${V}_{O}^{\cdot \cdot }$) for charge compensation (Eq. 3). Introducing Sr deficiency regenerates vacancies and partially elevates Fe valence via reactions (Eqs. 4, 5). Thereby, A-site vacancies counterbalance the Nb-induced vacancy reduction, modulating bulk transport and surface reactivity.

### Electrochemical Performance of Symmetrical Cells

The compatibility of electrode candidate materials with electrolyte components is critical for long-term stable cell operation. To examine the chemical compatibility of SF-based materials and BZCYYb electrolytes, D-SFN and BZCYYb were mixed and co-fired at 1100 °C for 10 h. The powder XRD results (Fig. S8) confirmed the absence of any new phases, indicating excellent compatibility.

**Fig. 4:**
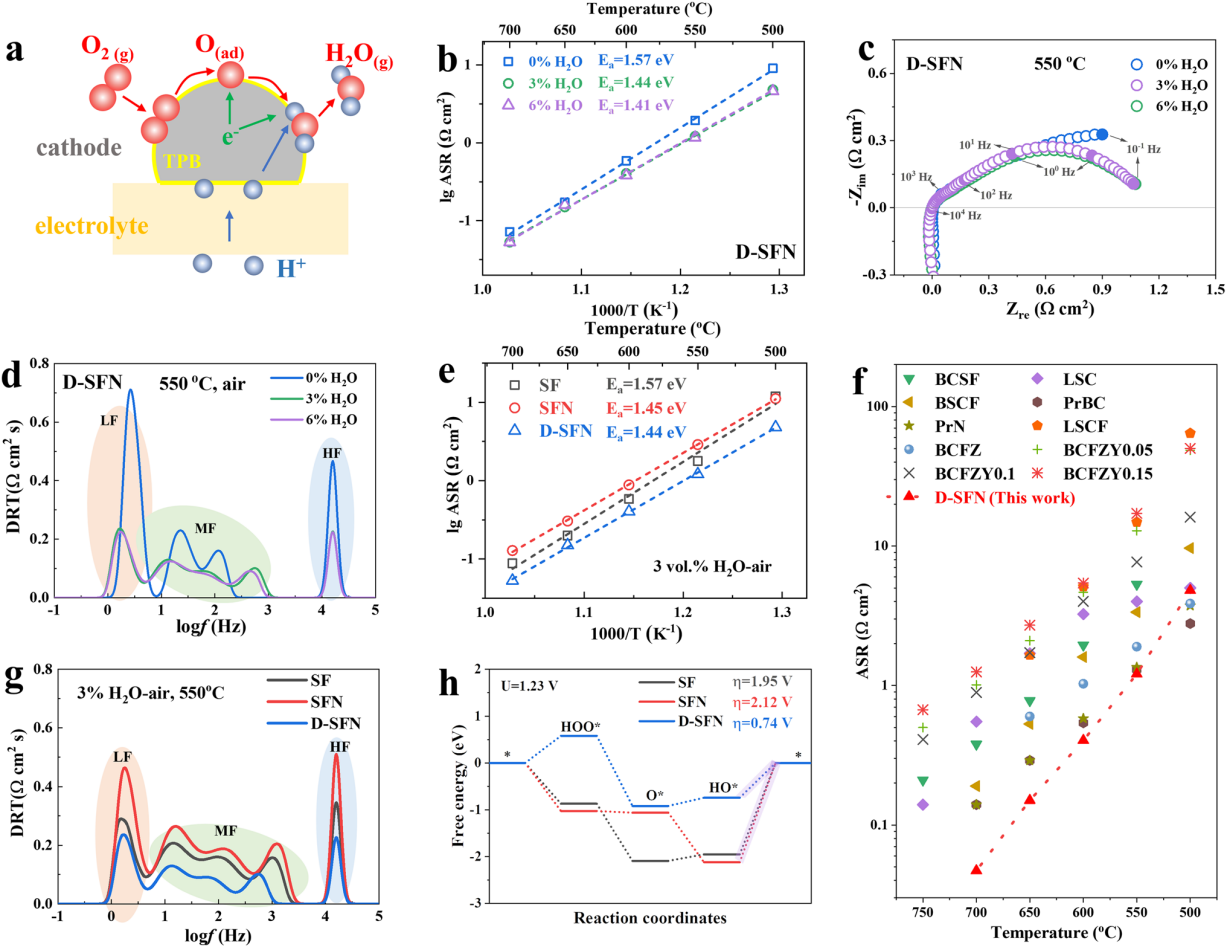
Electrochemical performance and reaction kinetics of the S_3−*y*_FN_*x*_ electrodes. **a** Schematic diagram of the ORR process in the humid air. **b** Arrhenius plots of D-SFN electrode under different *p*H_2_O. **c** Impact of *p*H_2_O on EIS of D-SFN electrode. **d** DRT revealing rate-determining steps. **e** Arrhenius plots quantifying ASR analyzed by S_3−*y*_FN_*x*_|BZCYYb|S_3−*y*_FN_*x*_ symmetrical cells. **f** Performance comparison of D-SFN electrode with the reported electrodes in the humid air. **g** DRT curves in the humid air. **h** Free energies of the ORR reaction on the surface of S_3−*y*_*FN*_*x*_

Different from the ORR in SOFC mode and OER in SOEC mode in the air electrode of traditional SOCs, due to the participation of water vapor, the air electrode of RePCC performs more complex ORR in PCFC mode and WOR in PCEC mode [51]. For illustration, Fig. 4a shows a schematic diagram of the ORR reaction occurring at the air electrode in PCFC mode, and the reverse process is WOR. The introduction of steam generates proton defects by promoting the hydration reaction between surface oxygen vacancies and water vapor, causing the three-phase boundary to expand from the original electrode–electrolyte interface under dry air (Fig. S9) to encompass the entire air electrode surface (Fig. 4a), playing a key role in enhancing ORR/WOR dynamics [63, 64].

To reveal the electrochemical performance of S_3-y_FN_x_ candidates as RePCC air electrodes, EIS tests were conducted on symmetrical cells under different air humidity (0, 3, and 6 vol%) and temperature (500–700 °C), and the Arrhenius curves of area specific resistance (ASR) are shown in Figs. 4b and S10. As the steam partial pressure in the air increases, the ASR and electrode reaction activation energy (*E*_a_) of the three electrode materials are significantly optimized, mainly because hydration generates proton defects, which effectively improves the bulk proton conductivity of the materials.

To further distinguish the ORR/WOR reaction processes of the D-SFN air electrode, the EIS curve (Fig. 4c) was obtained under various vapor pressures. The DRT method was employed to discern specific electrochemical sub-processes, leveraging the characteristic frequency response (10^5^–10^−1^ Hz) of the EIS curve [65, 66], as illustrated in Fig. 4d. Typically, low frequency (LF, 10^–1^–10^1^ Hz) peaks signify gas diffusion or surface adsorption/desorption steps. Mid-frequency (MF, 10^1^–10^4^ Hz) peaks correlate to surface oxygen exchange and/or bulk diffusion processes. High frequency (HF, 10^4^–10^5^ Hz) peaks represent charge transfer reactions. The peak area in a specific frequency range represents the polarization resistance of the corresponding electrochemical substep. It is noted that at different vapor partial pressures, the MF and LF peak areas constitute the main part of the entire DRT curve, indicating that gas diffusion, surface exchange, and bulk diffusion resistance are the main components of polarization resistance. After the introduction of water vapor, the MF and LF resistance dropped significantly, indicating that the introduction of water vapor significantly improved the diffusion of water, surface mass transfer, and bulk ion transport performance of the electrode [40].

In addition, the electrochemical performance of the three electrode materials was compared under the conventional operating atmosphere of the air electrode (3 vol% H_2_O-air) to evaluate the impact of Nb doping and A-site defects on the ORR/WOR reaction of the electrode. As shown in Fig. 4e, at 500–700 °C, the electrode reaction activation energy of SFN and D-SFN decreases significantly, indicating that the Nb substitution is beneficial to low-temperature operation. However, the incorporation of Nb reduces the electrochemical activity of SF electrodes, as can be seen from the increased polarization resistance. The further introduction of A-site Sr defects enables the D-SFN electrode to exhibit optimal ORR/WOR performance in the entire temperature range. At 550 °C, the ASR values of SF, SFN, and D-SFN were 1.781, 2.885, and 1.209 Ω cm^2^ respectively. To provide a more objective evaluation of the performance of the D-SFN electrode, Fig. 4f and Table S7 compare the ASR of D-SFN with that of high-performance air electrodes reported in the literature. The ASR of D-SFN is even significantly lower than many Co-based air electrodes, demonstrating outstanding electrocatalytic performance.

Besides, combined with DRT to further separate the electrode reaction process, as shown in Fig. 4g, the incorporation of Nb has adverse effects on both the surface mass transfer and the bulk diffusion process. The further introduction of Sr defects not only eliminates the adverse effects of Nb doping on the electrode reaction but also significantly reduces the resistance of all electrochemical sub-steps. The performance improvement can be attributed to the fact that the D-SFN material has more surface and bulk oxygen vacancies and presents the best surface exchange and bulk diffusion rates, which are key properties of the ORR/WOR reaction [8].

The ORR reaction process based on a four-electron mechanism further elucidates the ORR reaction mechanisms on the surfaces of three air electrodes [24, 67]. The ORR reaction-free energies on the surfaces of SF, Nb-substituted SFN, and A-site deficient D-SFN are depicted in Fig. 4h. The ORR on the air electrode surface comprises four steps. According to the ORR reaction barrier, it is evident that the rate-determining step for SF, SFN, and D-SFN electrode is located at the H_2_O formation step. For SF, the overpotential (η) is 1.95 V, for SFN, the overpotential is 2.12 V, and D-SFN exhibits a low η of 0.74 V, which is favorable for the rapid reaction kinetics of the air electrode.

### Stability Analysis of Symmetrical Cells

**Fig. 5:**
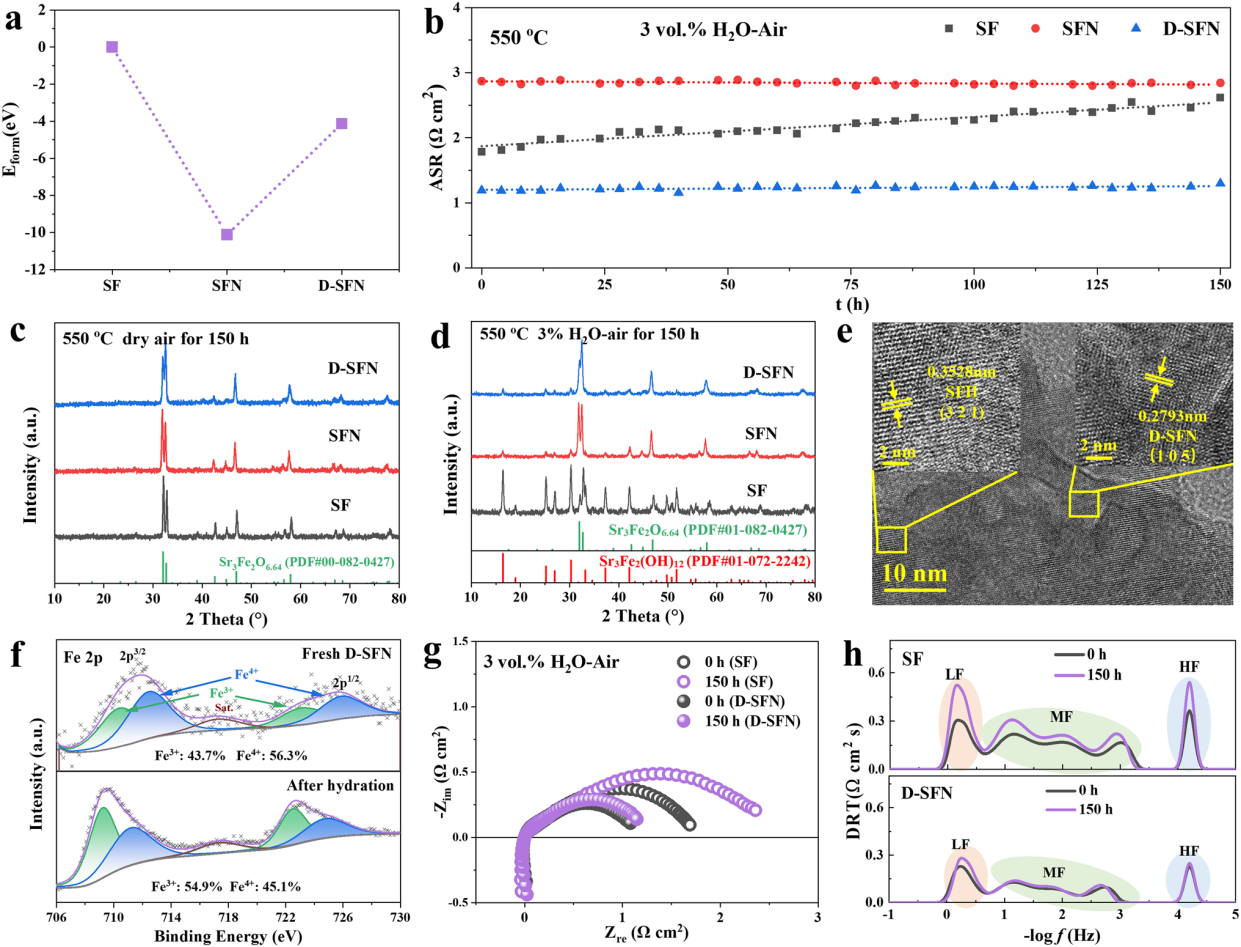
Stability assessments of S_3−*y*_FN_*x*_ electrodes. **a** Perovskite formation energy (*E*_form_) of S_3−*y*_FN_*x*_. **b** ASR evolution during humid air symmetrical cell testing. **c** powder XRD processed in dry air. **d** powder XRD processed in the humid air. **e** TEM of D-SFN powders treated in the humid air for 150 h. **f** Fe 2*p* XPS spectra of D-SFN before and after hydration for 150 h. **g** EIS evolution of SF and D-SFN electrode. **h** DRT analysis of EIS curves in **g**

Long-term polarization resistance tests were performed on symmetrical cells to further evaluate the stability of the D-SFN electrode material. To facilitate the explanation of the effects of Sr defects and Nb doping, the stability of SF and SFN materials was also checked. The formation energies of the three materials were initially calculated (Fig. 5a) and the calculation details can be found in the supporting information. Both SFN and D-SFN exhibited lower *E*_form_ than SF, indicating that the incorporation of Nb into the Fe site enhances the stability of the perovskite. The EIS tests were then conducted in 3% H_2_O-air at 550 °C for 150 h, and the ASR evolution was shown in Fig. 5b. SF electrode exhibited continuously increasing ASR indicating performance degradation over time, while Nb-doped SFN and D-SFN demonstrated negligible ASR changes, highlighting remarkably stable operation. This result confirms the the variation in *E*_form_ depicted in Fig. 5a, signifying that the introduction of Nb enhances the stability of the material.

To probe the origin of the performance divergence during long-term tests, the phase structure of the three materials in the air was analyzed. First, S_3−*y*_FN_*x*_ powders were treated at 550 °C for 150 h in dry air to explore the phase structure evolution. As shown in Fig. 5c, all materials maintain the initial phase structure, indicating that SF-based materials can maintain long-term phase structure stability in dry air. Phase stability was further explored under humid air for 150 h (Fig. 5d). SF material underwent significant phase decomposition, forming a large amount of Sr_3_Fe_2_(OH)_12_ (PDF#01-072-2242), which could be attributed to the extensive hydration-induced decomposition that SF underwent [68, 69]. The flexible SrO layer enables water incorporation, disrupting the parent lattice [70–72]. In contrast, only a small amount of the second phase appears in niobium-doped SFN and D-SFN, confirming their excellent structural stability.

The D-SFN powder, subjected to prolonged wet treatment, was collected for TEM analysis. As depicted in Fig. 5e, the surface of the particles reveals the (115) crystal plane spacing of the D-SFN phase and the (111) crystal plane spacing of the SFH phase. This suggests that the SFH phase forms on the surface of D-SFN after prolonged treatment, consistent with the results in Fig. 5d. Additionally, a comparison of the Fe 2*p* XPS spectra of D-SFN before and after wet treatment (Fig. 5f) demonstrates an increase in Fe^3+^ and a decrease in Fe^4+^, resulting in a decrease in the average oxidation state of Fe from + 3.56 to + 3.45. According to the literature, in the SFH phase, Fe ions coordinate directly with OH-, exhibiting a + 3-oxidation state [70]. The reduction in the average oxidation state of Fe after hydration also indirectly confirms the formation of the SFH phase.

EIS and DRT analysis (Fig. 5g, h) of the deteriorating SF electrode over time provides further evidence of continuously increasing LF and MF peak resistances. Since these revelations correlate to surface and bulk processes, excessive hydration likely hinders oxygen exchange and diffusion by occupying lattice oxygen sites and vacancies [73]. While initially enhancing performance, unrestrained hydration can thus degrade long-term stability. In contrast, Nb-doped D-SFN maintains high activity alongside exceptional steam stability by stabilizing the lattice oxygen to prevent undesirable hydroxide formation. Ultimately, elucidating the intricate effects of composition and hydration on long-term RePCC electrode performance provides invaluable guidance for designing highly stable and active air electrode materials.

### Performance and Durability of RePCC

**Fig. 6:**
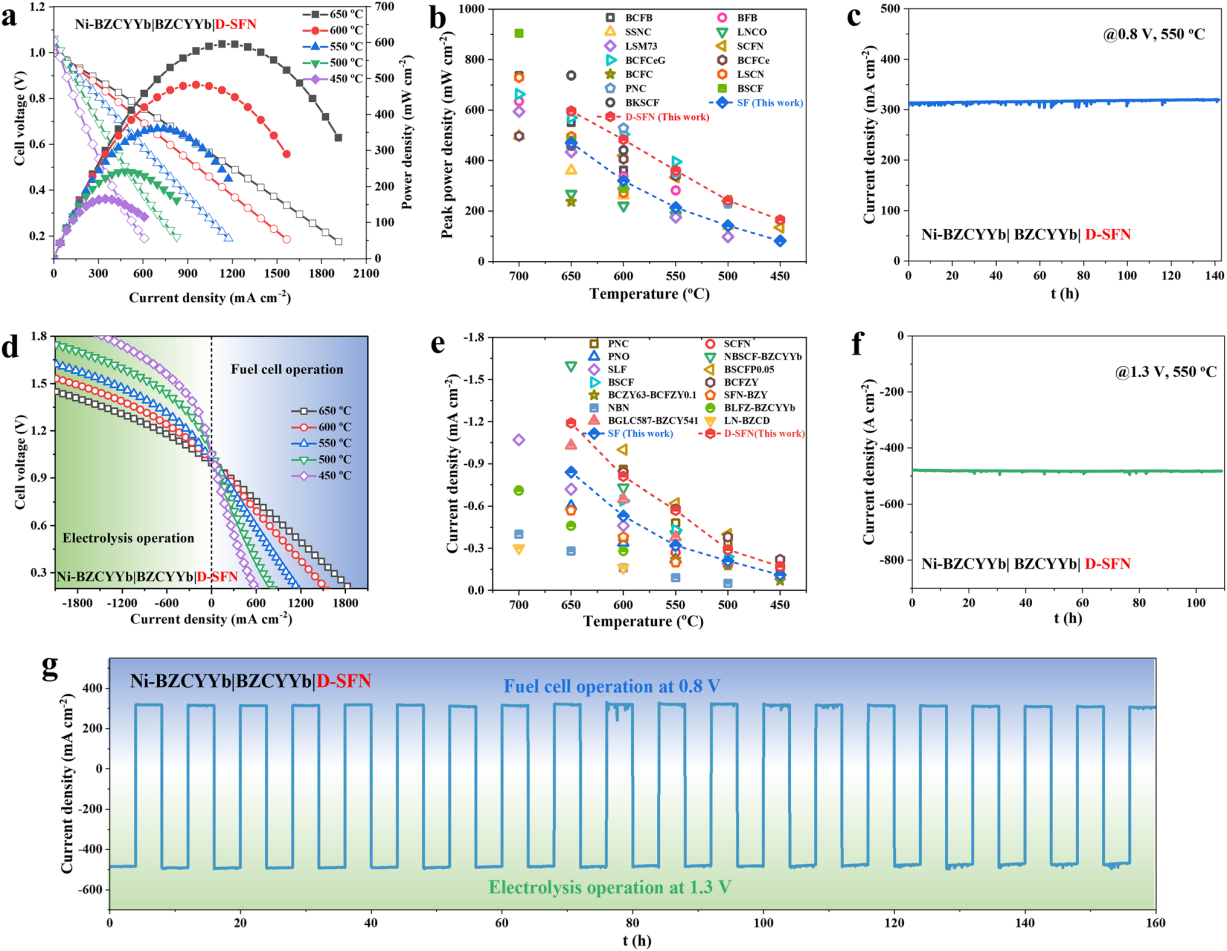
Electrochemical performance of Ni-BZCYYb|BZCYYb|D-SFN single cell. **a** Fuel cell power density curves under H_2_/ humidified air. **b** Benchmarking peak power density against recently reported high-performance PCFCs. **c** Long-term stability under PCFC mode (at 0.8 V). **d** Polarization curves exhibiting high current density under H_2_/humidified air. **e** Benchmarking electrolysis performance against state-of-the-art PCECs at 1.3 V. **f** Durability under PCEC mode (at 1.3 V). **g** Outstanding cyclic stability over 160 h with 23 reversal cycles between fuel cell (0.8 V) and electrolysis (1.3 V) modes

To evaluate the promising D-SFN air electrode candidate material in a practical RePCC device, a Ni-BZCYYb fuel electrode-supported single cell was fabricated comprising a ~ 23 μm electrolyte and ~ 10 μm D-SFN air electrode (Fig. S11). A single cell with the same configuration using SF as the air electrode was also prepared, which has the same electrolyte thickness as the single cell with the D-SFN air electrode (Fig. S11). D-SFN based fuel cell (PCFC) *i-V-P* curves (Fig. 6a) exhibit exceptional peak power densities (PPD) of 596, 483, 361, 242, and 165 mW cm^−2^ from 650 to 450 °C, respectively, using H_2_-humidified air feeds. This significantly outperforms the same configuration of a single cell with the SF air electrode (Fig. S12), directly confirming the optimized ORR electrocatalytic performance of the D-SFN air electrode. The PPDs surpass numerous reported PCFCs employing advanced cathodes (Fig. 6b, Table S8), and the cell operates stably for approximately 142 h under 0.8 V (Fig. 6c), highlighting the potential of D-SFN for air electrodes in PCFCs.

Besides, RePCCs can not only work in PCFC mode, but also PCEC mode, so it is vital to investigate the performance of the D-SFN air electrode candidate in electrolysis mode (PCEC). Under electrolysis mode at 1.3 V (Fig. 6d), high current densities of − 0.17 to − 1.19 A cm^−2^ were achieved from 450 to 650 °C, exhibiting much higher current densities than electrolysis cell with the SF air electrode (Fig. S12). The performance is even comparable to cells with top-tier air electrodes (Fig. 6e, Table S9). Excellent 109-h stability at 1.3 V (Fig. 6f) demonstrates negligible degradation. The *i-V* curve and electrolysis stability test prove the excellent OER activity and durability of the D-SFN air electrode.

In addition, considering the working scenario of RePCCs combined with renewable energy, it is critical to examine the reversibility of the D-SFN air electrode, because the cell needs to switch between PCFC and PCEC modes intermittently [74]. The cell underwent alternating cycles of operation in PCFC mode (at 0.8 V) and PCEC mode (at 1.3 V) for 4 h each, totaling 20 cycles and lasting 160 h (Fig. 6g). Despite repeated oxidation and reduction, only slight performance loss occurred in the cell, verifying the excellent stability of the electrode and cell reversibility.

By extensively evaluating the optimized D-SFN air electrode material in a single-cell configuration, this work substantiates its high activity for ORR/WOR electrocatalysis along with excellent stability for practical RePCC devices. The exceptional outputs highlight the efficacy of the co-substitution approach in developing optimized electrode materials.

## Conclusions

This work demonstrates a simple A/B-sites co-substitution strategy to develop RP-type ferrites RePCC air electrodes with improved activity and stability. Introducing A-site Sr deficiency and Nb doping in RP-type SF air electrode, realizing a rationally engineered D-SFN composition. Nb incorporation enhances structural stability but reduces oxygen vacancies and transport. Counteracting A-site deficiency recovers crucial vacancies to promote surface reactions and bulk diffusion, thereby optimizing electrocatalytic activity and durability. Consequently, symmetrical cell studies verify D-SFN's remarkably high performance and negligible degradation during 150 h humid air operation. When implemented in single cells, the D-SFN-based cell achieved an outstanding 596 mW cm^−2^ PPD in fuel cell mode at 650 °C along with a substantial − 1.19 A cm^−2^ current density at 1.3 V in electrolysis mode using hydrogen and humidified air feeds. Furthermore, exceptional stability was demonstrated for 160 h and 20 cycles during fuel cell-electrolysis cycling mode. Through intergrating doping and deficiency modification, this work provides critical insights into balancing activity and stability in high-performance RePCC air electrodes. The D-SFN material exemplifies the promise of this approach for developing durable and active air electrodes for efficient renewable energy storage and conversion.

## Supplementary Information

Below is the link to the electronic supplementary material.

Supplementary file1 (PDF 1961 kb)

## References

[1] D. Bidwell, Thinking through participation in renewable energy decisions. Nat. Energy **1**, 16051 (2016). 10.1038/nenergy.2016.51

[2] S. Zhao, T. Liu, Y. Dai, J. Wang, Y. Wang et al., Pt/C as a bifunctional ORR/iodide oxidation reaction (IOR) catalyst for Zn-air batteries with unprecedentedly high energy efficiency of 7.65%. Appl. Catal. B Environ. **320**, 121992 (2023). 10.1016/j.apcatb.2022.121992

[3] P.P. Lopes, V.R. Stamenkovic, Past, present, and future of lead-acid batteries. Science **369**, 923–924 (2020). 10.1126/science.abd3352 32820114 10.1126/science.abd3352

[4] B. Gangaja, S. Nair, D. Santhanagopalan, Surface-engineered Li_4_Ti_5_O_12_ nanostructures for high-power Li-ion batteries. Nano-Micro Lett. **12**, 30 (2020). 10.1007/s40820-020-0366-x 10.1007/s40820-020-0366-x PMC7770703 34138269

[5] H. Wang, J. Gao, C. Chen, W. Zhao, Z. Zhang et al., PtNi-W/C with atomically dispersed tungsten sites toward boosted ORR in proton exchange membrane fuel cell devices. Nano-Micro Lett. **15**, 143 (2023). 10.1007/s40820-023-01102-9 10.1007/s40820-023-01102-9 PMC10236083 37266746

[6] W. Lv, Z. Tong, Y.-M. Yin, J. Yin, Z.-F. Ma, Novel nano-composites SDC-LiNaSO_4_ as functional layer for ITSOFC. Nano-Micro Lett. **7**, 268–275 (2015). 10.1007/s40820-015-0038-4 10.1007/s40820-015-0038-4 PMC6223898 30464971

[7] C. Duan, R. Kee, H. Zhu, N. Sullivan, L. Zhu et al., Highly efficient reversible protonic ceramic electrochemical cells for power generation and fuel production. Nat. Energy **4**, 230–240 (2019). 10.1038/s41560-019-0333-2

[8] Y. Song, J. Liu, Y. Wang, D. Guan, A. Seong et al., Nanocomposites: a new opportunity for developing highly active and durable bifunctional air electrodes for reversible protonic ceramic cells. Adv. Energy Mater. **11**, 2101899 (2021). 10.1002/aenm.202101899

[9] W. Zhang, Y. Zhou, X. Hu, Y. Ding, J. Gao et al., A synergistic three-phase, triple-conducting air electrode for reversible proton-conducting solid oxide cells. ACS Energy Lett. **8**, 3999–4007 (2023). 10.1021/acsenergylett.3c01251 37854047 10.1021/acsenergylett.3c01251 PMC10580316

[10] Z. Liu, Z. Tang, Y. Song, G. Yang, W. Qian et al., High-entropy perovskite oxide: a new opportunity for developing highly active and durable air electrode for reversible protonic ceramic electrochemical cells. Nano-Micro Lett. **14**, 217 (2022). 10.1007/s40820-022-00967-6 10.1007/s40820-022-00967-6 PMC9646682 36352041

[11] A. Magrasó, R. Haugsrud, M. Segarra, T. Norby, Defects and transport in Gd-doped BaPrO_3_. J. Electroceram. **23**, 80–88 (2009). 10.1007/s10832-008-9541-z

[12] S. Choi, C.J. Kucharczyk, Y. Liang, X. Zhang, I. Takeuchi et al., Exceptional power density and stability at intermediate temperatures in protonic ceramic fuel cells. Nat. Energy **3**, 202–210 (2018). 10.1038/s41560-017-0085-9

[13] N. Wang, C. Tang, L. Du, R. Zhu, L. Xing et al., Advanced cathode materials for protonic ceramic fuel cells: Recent progress and future perspectives. Adv. Energy Mater. **12**, 2201882 (2022). 10.1002/aenm.202201882

[14] R. Zohourian, R. Merkle, G. Raimondi, J. Maier, Mixed-conducting perovskites as cathode materials for protonic ceramic fuel cells: understanding the trends in proton uptake. Adv. Funct. Mater. **28**(35), 1801241 (2018). 10.1002/adfm.201801241

[15] Z. Wang, W. Yang, S.P. Shafi, L. Bi, Z. Wang et al., A high performance cathode for proton conducting solid oxide fuel cells. J. Mater. Chem. A **3**, 8405–8412 (2015). 10.1039/c5ta00391a

[16] D. Huan, N. Shi, L. Zhang, W. Tan, Y. Xie et al., New, efficient, and reliable air electrode material for proton-conducting reversible solid oxide cells. ACS Appl. Mater. Interfaces **10**, 1761–1770 (2018). 10.1021/acsami.7b16703 29282974 10.1021/acsami.7b16703

[17] N. Tarasova, I. Animitsa, A. Galisheva, V. Pryakhina, Protonic transport in the new phases BaLaIn_0.9_M_0.1_O_4.05_ (M=Ti, Zr) with Ruddlesden–Popper structure. Solid State Sci. **101**, 106121 (2020). 10.1016/j.solidstatesciences.2020.106121

[18] A. Niemczyk, R. Merkle, J. Maier, K. Świerczek, Defect chemistry and proton uptake of La_2__−__*x*_Sr_*x*_NiO_4±δ_ and La_2__−__*x*_Ba_*x*_NiO_4±δ_ Ruddlesden-Popper phases. J. Solid State Chem. **306**, 122731 (2022). 10.1016/j.jssc.2021.122731

[19] Z.L. Moreno Botello, A. Montenegro, N. Grimaldos Osorio, M. Huvé, C. Pirovano et al., Pure and Zr-doped YMnO_3+__*δ*_ as a YSZ-compatible SOFC cathode: A combined computational and experimental approach. J. Mater. Chem. A **7**, 18589–18602 (2019). 10.1039/c9ta04912f

[20] J. Xu, H. Cai, G. Hao, L. Zhang, Z. Song et al., Characterization of high–valence Mo–doped PrBaCo_2_O^5+^ cathodes for IT–SOFCs. J. Alloys Compd. **842**, 155600 (2020). 10.1016/j.jallcom.2020.155600

[21] C. Lu, R. Ren, Z. Zhu, G. Pan, G. Wang et al., BaCo_0.4_Fe_0.4_Nb_0.1_Sc_0.1_O_3__−__δ_ perovskite oxide with super hydration capacity for a high-activity proton ceramic electrolytic cell oxygen electrode. Chem. Eng. J. **472**, 144878 (2023). 10.1016/j.cej.2023.144878

[22] J. Wang, M. Saccoccio, D. Chen, Y. Gao, C. Chen et al., The effect of A-site and B-site substitution on BaFeO_3–__*δ*_: An investigation as a cathode material for intermediate-temperature solid oxide fuel cells. J. Power. Sources **297**, 511–518 (2015). 10.1016/j.jpowsour.2015.08.016

[23] F. Dong, Y. Chen, R. Ran, D. Chen, M.O. Tadé et al., BaNb_0.05_Fe_0.95_O_3–δ_ as a new oxygen reduction electrocatalyst for intermediate temperature solid oxide fuel cells. J. Mater. Chem. A **1**, 9781 (2013). 10.1039/c3ta11447c

[24] Z. Wang, Y. Wang, J. Wang, Y. Song, M.J. Robson et al., Rational design of perovskite ferrites as high-performance proton-conducting fuel cell cathodes. Nat. Catal. **5**, 777–787 (2022). 10.1038/s41929-022-00829-9

[25] X. Chen, N. Yu, I.T. Bello, D. Guan, Z. Li et al., Facile anion engineering: A pathway to realizing enhanced triple conductivity in oxygen electrodes for reversible protonic ceramic electrochemical cells. Energy Storage Mater. **63**, 103056 (2023). 10.1016/j.ensm.2023.103056

[26] T.H. Wan, M. Saccoccio, C. Chen, F. Ciucci, Influence of the discretization methods on the distribution of relaxation times deconvolution: Implementing radial basis functions with DRTtools. Electrochim. Acta **184**, 483–499 (2015). 10.1016/j.electacta.2015.09.097

[27] J. Liu, F. Ciucci, The Gaussian process distribution of relaxation times: A machine learning tool for the analysis and prediction of electrochemical impedance spectroscopy data. Electrochim. Acta **331**, 135316 (2020). 10.1016/j.electacta.2019.135316

[28] G. Kresse, J. Furthmüller, Efficiency of ab-initio total energy calculations for metals and semiconductors using a plane-wave basis set. Comput. Mater. Sci. **6**, 15–50 (1996). 10.1016/0927-0256(96)00008-0 10.1103/physrevb.54.11169 9984901

[29] G. Kresse, J. Furthmüller, Efficient iterative schemes for *ab initio* total-energy calculations using a plane-wave basis set. Phys. Rev. B Condens. Matter **54**, 11169–11186 (1996). 10.1103/physrevb.54.11169 9984901 10.1103/physrevb.54.11169

[30] J.P. Perdew, K. Burke, M. Ernzerhof, Generalized gradient approximation made simple. Phys. Rev. Lett. **77**, 3865–3868 (1996). 10.1103/PhysRevLett.77.3865 10062328 10.1103/PhysRevLett.77.3865

[31] A. Hagiwara, N. Hobara, K. Takizawa, K. Sato, H. Abe et al., Preparation and evaluation of mechanochemically fabricated LSM/ScSZ composite materials for SOFC cathodes. Solid State Ion. **177**, 2967–2977 (2006). 10.1016/j.ssi.2006.08.021

[32] D. Huan, L. Zhang, K. Zhu, X. Li, N. Shi et al., Oxygen vacancy-engineered cobalt-free Ruddlesden–Popper cathode with excellent CO_2_ tolerance for solid oxide fuel cells. J. Power. Sources **497**, 229872 (2021). 10.1016/j.jpowsour.2021.229872

[33] S. Jiang, J. Sunarso, W. Zhou, J. Shen, R. Ran et al., Cobalt-free SrNb_x_Fe_1–x_O_3–δ_ (x=0.05, 0.1 and 0.2) perovskite cathodes for intermediate temperature solid oxide fuel cells. J. Power. Sources **298**, 209–216 (2015). 10.1016/j.jpowsour.2015.08.063

[34] D.O. Idisi, E.L. Meyer, E.M. Benecha, The role of iron oxide on the electronic and electrical properties of nitrogenated reduced graphene oxide: experimental and density functional theory approach. J. Mater. Sci. Mater. Electron. **35**, 192 (2024). 10.1007/s10854-024-11947-4

[35] T.T. Pham, T.N. Pham, V. Chihaia, Q.A. Vu, T.T. Trinh et al., How do the doping concentrations of N and B in graphene modify the water adsorption? RSC Adv. **11**, 19560–19568 (2021). 10.1039/d1ra01506k 35479230 10.1039/d1ra01506k PMC9033564

[36] H. Zhang, Y. Gao, H. Xu, D. Guan, Z. Hu et al., Combined corner-sharing and edge-sharing networks in hybrid nanocomposite with unusual lattice-oxygen activation for efficient water oxidation. Adv. Funct. Mater. **32**, 2207618 (2022). 10.1002/adfm.202207618

[37] A. Fujimori, A.E. Bocquet, T. Saitoh, T. Mizokawa, Electronic structure of 3d transition metal compounds: systematic chemical trends and multiplet effects. J. Electron Spectrosc. Relat. Phenom. **62**, 141–152 (1993). 10.1016/0368-2048(93)80011-a

[38] A. Atkinson, S. Barnett, R.J. Gorte, J.T.S. Irvine, A.J. McEvoy et al., Advanced anodes for high-temperature fuel cells. Nat. Mater. **3**, 17–27 (2004). 10.1038/nmat1040 14704781 10.1038/nmat1040

[39] M.D. Gross, J.M. Vohs, R.J. Gorte, Recent progress in SOFC anodes for direct utilization of hydrocarbons. J. Mater. Chem. **17**, 3071–3077 (2007). 10.1039/B702633A

[40] M. Liang, Y. Zhu, Y. Song, D. Guan, Z. Luo et al., A new durable surface nanoparticles-modified perovskite cathode for protonic ceramic fuel cells from selective cation exsolution under oxidizing atmosphere. Adv. Mater. **34**, e2106379 (2022). 10.1002/adma.202106379 34962667 10.1002/adma.202106379

[41] X. Zhou, N. Hou, T. Gan, L. Fan, Y. Zhang et al., Enhanced oxygen reduction reaction activity of BaCe_0.2_Fe_0.8_O_3–δ_ cathode for proton-conducting solid oxide fuel cells via Pr-doping. J. Power. Sources **495**, 229776 (2021). 10.1016/j.jpowsour.2021.229776

[42] W. Xia, Q. Li, L. Sun, L. Huo, H. Zhao, Enhanced electrochemical performance and CO_2_ tolerance of Ba_0.95_La_0.05_Fe_0.85_Cu_0.15_O_3_- as Fe-based cathode electrocatalyst for solid oxide fuel cells. J. Eur. Ceram. Soc. **40**, 1967–1974 (2020). 10.1016/j.jeurceramsoc.2020.01.039

[43] X. Xu, H. Wang, M. Fronzi, X. Wang, L. Bi et al., Tailoring cations in a perovskite cathode for proton-conducting solid oxide fuel cells with high performance. J. Mater. Chem. A **7**, 20624–20632 (2019). 10.1039/c9ta05300j

[44] S.L. Millican, A.M. Deml, M. Papac, A. Zakutayev, R. O’Hayre et al., Predicting oxygen off-stoichiometry and hydrogen incorporation in complex perovskite oxides. Chem. Mater. **34**, 510–518 (2022). 10.1021/acs.chemmater.0c04765

[45] H. Li, J. Li, X. Wang, C. Xie, Y. Wang et al., Electrochemical performance and enhancement of hydration kinetics on BaCo_0.7_Fe_0.2_Zr_0.1_O_3–δ_ cathode for protonic ceramic fuel cells. ACS Appl. Energy Mater. **6**, 8966–8975 (2023). 10.1021/acsaem.3c01698

[46] W. Zhang, H. Muroyama, Y. Mikami, Q. Liu, X. Liu et al., Effectively enhanced oxygen reduction activity and stability of triple-conducting composite cathodes by strongly interacting interfaces for protonic ceramic fuel cells. Chem. Eng. J. **461**, 142056 (2023). 10.1016/j.cej.2023.142056

[47] Y. Lee, J. Kleis, J. Rossmeisl, D. Morgan, Ab initio energetics of LaBO_3_ (001) (B=Mn, Fe Co, and Ni) for solid oxide fuel cell cathodes. Phys. Rev. B **80**(22), 224101 (2009). 10.1103/PhysRevB.80.224101

[48] D. Huan, L. Zhang, X. Li, Y. Xie, N. Shi et al., A durable ruddlesden-popper cathode for protonic ceramic fuel cells. ChemSusChem **13**, 4994–5003 (2020). 10.1002/cssc.202001168 32671967 10.1002/cssc.202001168

[49] J. Wang, K.Y. Lam, M. Saccoccio, Y. Gao, D. Chen et al., Ca and in Co-doped BaFeO_3–δ_ as a cobalt-free cathode material for intermediate-temperature solid oxide fuel cells. J. Power. Sources **324**, 224–232 (2016). 10.1016/j.jpowsour.2016.05.089

[50] S. Wang, J. Zan, W. Qiu, D. Zheng, F. Li et al., Evaluation of perovskite oxides LnBaCo_2_O_5+__*δ*_ (Ln=La, Pr, Nd and Sm) as cathode materials for IT-SOFC. J. Electroanal. Chem. **886**, 115144 (2021). 10.1016/j.jelechem.2021.115144

[51] Y. Song, Y. Chen, W. Wang, C. Zhou, Y. Zhong et al., Self-assembled triple-conducting nanocomposite as a superior protonic ceramic fuel cell cathode. Joule **3**, 2842–2853 (2019). 10.1016/j.joule.2019.07.004

[52] J. Kim, A. Jun, J. Shin, G. Kim, Effect of Fe doping on layered GdBa_0.5_Sr_0.5_Co_2_O_5+δ_ perovskite cathodes for intermediate temperature solid oxide fuel cells. J. Am. Ceram. Soc. **97**(2), 651–656 (2014). 10.1111/jace.12692

[53] M. Gou, R. Ren, W. Sun, C. Xu, X. Meng et al., Nb-doped Sr_2_Fe_1.5_Mo_0.5_O_6__−__δ_ electrode with enhanced stability and electrochemical performance for symmetrical solid oxide fuel cells. Ceram. Int. **45**, 15696–15704 (2019). 10.1016/j.ceramint.2019.03.130

[54] S. Yun, J. Yu, W. Lee, H. Lee, W.-S. Yoon, Achieving structural stability and enhanced electrochemical performance through Nb-doping into Li- and Mn-rich layered cathode for lithium-ion batteries. Mater. Horiz. **10**, 829–841 (2023). 10.1039/d2mh01254e 36597945 10.1039/d2mh01254e

[55] Y. Zhang, B. Chen, D. Guan, M. Xu, R. Ran et al., Thermal-expansion offset for high-performance fuel cell cathodes. Nature **591**, 246–251 (2021). 10.1038/s41586-021-03264-1 33692558 10.1038/s41586-021-03264-1

[56] Y. Fu, A. Subardi, M. Hsieh, W. Chang, Electrochemical properties of La_0.5_Sr_0.5_Co_0.8_M_0.2_O_3__−__δ_ (M=Mn, Fe, Ni, Cu) perovskite cathodes for IT-SOFCs. J. Am. Ceram. Soc. **99**(4), 1345–1352 (2016). 10.1111/jace.14127

[57] Y. Wan, Y. Xing, Y. Li, D. Huan, C. Xia, Thermal cycling durability improved by doping fluorine to PrBaCo_2_O_5+__*δ*_ as oxygen reduction reaction electrocatalyst in intermediate-temperature solid oxide fuel cells. J. Power. Sources **402**, 363–372 (2018). 10.1016/j.jpowsour.2018.09.065

[58] Y. Wang, F. Jin, X. Hao, B. Niu, P. Lyu et al., B-site-ordered Co-based double perovskites Sr_2_Co1–Nb FeO^5+^ as active and stable cathodes for intermediate-temperature solid oxide fuel cells. J. Alloys Compd. **829**, 154470 (2020). 10.1016/j.jallcom.2020.154470

[59] B. Wei, Z. Lü, X. Huang, J. Miao, X. Sha et al., Crystal structure, thermal expansion and electrical conductivity of perovskite oxides Ba_*x*_Sr_1–__*x*_Co_0.8_Fe_0.2_O_3–δ_ (0.3≤*x*≤0.7). J. Eur. Ceram. Soc. **26**, 2827–2832 (2006). 10.1016/j.jeurceramsoc.2005.06.047

[60] H. An, H.-W. Lee, B.-K. Kim, J.-W. Son, K.J. Yoon et al., A 5 × 5 cm^2^ protonic ceramic fuel cell with a power density of 1.3 W cm^2^ at 600 °C. Nat. Energy **3**, 870–875 (2018). 10.1038/s41560-018-0230-0

[61] Y. Hou, L. Wang, L. Bian, Q. Zhang, L. Chen et al., Effect of high-valence elements doping at B site of La_0.5_Sr_0.5_FeO_3__−__δ_. Ceram. Int. **48**, 4223–4229 (2022). 10.1016/j.ceramint.2021.10.214

[62] I.T. Bello, S. Zhai, Q. He, C. Cheng, Y. Dai et al., Materials development and prospective for protonic ceramic fuel cells. Int. J. Energy Res. **46**, 2212–2240 (2022). 10.1002/er.7371

[63] K.-C. Lee, M.-B. Choi, D.-K. Lim, B. Singh, S.-J. Song, Effect of humidification on the performance of intermediate-temperature proton conducting ceramic fuel cells with ceramic composite cathodes. J. Power. Sources **232**, 224–233 (2013). 10.1016/j.jpowsour.2013.01.001

[64] R. Ren, Z. Wang, X. Meng, X. Wang, C. Xu et al., Tailoring the oxygen vacancy to achieve fast intrinsic proton transport in a perovskite cathode for protonic ceramic fuel cells. ACS Appl. Energy Mater. **3**, 4914–4922 (2020). 10.1021/acsaem.0c00486

[65] R. Ren, Z. Wang, C. Xu, W. Sun, J. Qiao et al., Tuning the defects of the triple conducting oxide BaCo_0.4_Fe_0.4_Zr_0.1_Y_0.1_O_3–δ_ perovskite toward enhanced cathode activity of protonic ceramic fuel cells. J. Mater. Chem. A **7**, 18365–18372 (2019). 10.1039/c9ta04335g

[66] Y. Li, Y. Tian, J. Li, J. Pu, B. Chi, Sr-free orthorhombic perovskite Pr_0.8_Ca_0.2_Fe_0.8_Co_0.2_O_3–δ_ as a high-performance air electrode for reversible solid oxide cell. J. Power. Sources **528**, 231202 (2022). 10.1016/j.jpowsour.2022.231202

[67] P. Yao, J. Zhang, Q. Qiu, G. Li, Y. Zhao et al., Design of a perovskite oxide cathode for a protonic ceramic fuel cell. Ceram. Int. **50**, 2373–2382 (2024). 10.1016/j.ceramint.2023.11.015

[68] M.F. Hoedl, D. Gryaznov, R. Merkle, E.A. Kotomin, J. Maier, Interdependence of oxygenation and hydration in mixed-conducting (Ba, Sr)FeO_3–__*δ*_ perovskites studied by density functional theory. J. Phys. Chem. C **124**, 11780–11789 (2020). 10.1021/acs.jpcc.0c01924

[69] R. Merkle, M.F. Hoedl, G. Raimondi, R. Zohourian, J. Maier, Oxides with mixed protonic and electronic conductivity. Annu. Rev. Mater. Res. **51**, 461–493 (2021). 10.1146/annurev-matsci-091819-010219

[70] M. Matvejeff, M. Lehtimäki, A. Hirasa, Y.-H. Huang, H. Yamauchi et al., New water-containing phase derived from the Sr_3_Fe_2_O_7__−__*δ*_ phase of the Ruddlesden−Popper structure. Chem. Mater. **17**, 2775–2779 (2005). 10.1021/cm050106z

[71] K.V. Zakharchuk, A.A. Yaremchenko, D.P. Fagg, Electrical properties and thermal expansion of strontium aluminates. J. Alloys Compd. **613**, 232–237 (2014). 10.1016/j.jallcom.2014.05.225

[72] P.R. Slater, C. Greaves, The ionic conductivity of proton containing garnets and their decomposition products. Solid State Ion. **53–56**, 989–992 (1992). 10.1016/0167-2738(92)90281-s

[73] C. Zhou, D. Liu, M. Fei, X. Wang, R. Ran et al., Cathode water management towards improved performance of protonic ceramic fuel cells. J. Power. Sources **556**, 232403 (2023). 10.1016/j.jpowsour.2022.232403

[74] D. Zou, Y. Yi, Y. Song, D. Guan, M. Xu et al., The BaCe_0.16_Y_0.04_Fe_0.8_O_3–δ_ nanocomposite: a new high-performance cobalt-free triple-conducting cathode for protonic ceramic fuel cells operating at reduced temperatures. J. Mater. Chem. A **10**, 5381–5390 (2022). 10.1039/d1ta10652j

