# Supplementary material for: Rational Design of Ruddlesden–Popper Perovskite Ferrites as Air Electrode for Highly Active and Durable Reversible Protonic Ceramic Cells
Source: Nanomicro Lett. 2024 Apr 22;16:177. doi: 10.1007/s40820-024-01397-2 (PMC11035539; doi:10.1007/s40820-024-01397-2)
Supplement: Supplementary file 1 — Supplementary file1 (PDF 1961 kb) [file 40820_2024_1397_MOESM1_ESM.pdf]

Supporting Information for

Rational Design of Ruddlesden-Popper Perovskite Ferrites as Air Electrode for Highly Active and Durable Reversible Protonic Ceramic Cells

Na Yu[1], Idris Temitope Bello[1], Xi Chen[1], Tong Liu[1,2], Zheng Li[1], Yufei Song[3,*], Meng Ni[1,*]

[1] Department of Building and Real Estate, Research Institute for Sustainable Urban Development (RISUD) and Research Institute for Smart Energy (RISE), The Hong Kong Polytechnic University, Hung Hom, Hong Kong, Kowloon, P. R. China

[2] The Hong Kong Polytechnic University Shenzhen Research Institute, Shenzhen, Guangdong, 518057, P. R. China

[3] Department of Mechanical and Aerospace Engineering, The Hong Kong University of Science and Technology, Clear Water Bay, Hong Kong, P. R. China

***Corresponding authors. E-mail: yufeisong@ust.hk (Yufei Song); meng.ni@polyu.edu.hk (Meng Ni)

S1 Experimental Details

S1.1 Iodometric Titration Test

In this experimental titration, 0.1g of the powder sample was completely dissolved using a concentrated HCl solution (6 mol L^{-1}), along with excess KI powder. Subsequently, the resulting solution was diluted with water to maintain a weakly acidic pH (pH=6). The solution was titrated with fresh standard $Na_2S_2O_3$ solution (0.05 mol L^{-1}) twice, and near the titration endpoint, a fresh starch indicator was introduced to indicate the endpoint. The average volume of consumed $Na_2S_2O_3$ solution was calculated from the two titration experiments to determine the valence state of the Fe element and the concentration of oxygen vacancies.

S1.2 Electrical Conductivity Test

The electrical conductivity of the $S_{3-y}FN_x$ was examined by the DC four-probe method [S1, S2]. Dense bar samples were prepared with silver wires affixed as current collectors. A constant current of 100 mA was applied to the sample, while the voltage was recorded with a digital source meter (Keithley 2440). The conductivity (σ) of the material was calculated using the following Eqn. (S1):

$$\sigma = \frac{1}{\rho} = \frac{I}{U}\frac{l_2}{l_1 l_3} \qquad \text{(S1)}$$

where ρ is the resistivity, Ω m, I and U are the output current signal, A; U is the output voltage signals in A and V, respectively, and l_2, l_1, and l_3 are the length, width, and thickness of the bar sample in cm.

The electrical conductivity relaxation (ECR) test was done using the same configuration. The sample was placed in a 21% O_2-71% N_2 atmosphere. Once the output signal was stabilized, the atmosphere was quickly switched to 10% O_2-90% N_2 until it was stabilized again. The corresponding relaxation curves were recorded and converted into normalized conductivity (g(t)) using the expression (S2):

$$g(t) = \frac{\sigma_t - \sigma_0}{\sigma_\infty - \sigma_0}$$

$$= 1 - \sum_{n=1}^{\infty}\sum_{m=1}^{\infty}\sum_{p=1}^{\infty} \frac{2C_1^2 \exp\left(-\alpha_{1n}^2 D_{chem}\frac{t}{l_1^2}\right)}{\alpha_{1n}^2(\alpha_{1n}^2+C_1^2+C_1)} \cdot \frac{2C_2^2 \exp\left(-\alpha_{2m}^2 D_{chem}\frac{t}{l_2^2}\right)}{\alpha_{2m}^2(\alpha_{2m}^2+C_2^2+C_2)} \cdot \frac{2C_3^2 \exp\left(-\alpha_{3p}^2 D_{chem}\frac{t}{l_3^2}\right)}{\alpha_{3p}^2(\alpha_{3p}^2+C_3^2+C_3)} \quad \text{(S2)}$$

where σ_0, σ_t, and σ_∞ represent the conductivity at t=0, t=t, and t=∞ in S cm^{-1}. D_{chem} represents the chemical bulk diffusion coefficient of the material (cm^2 s^{-1}), while α_{1n}, α_{2m}, α_{3p} denote the nth, mth, and pth roots of the transcendental equation, respectively. Further details of the fitting procedure can be found in the literature [S3, S4]. In this study, the relaxation curves were fitted using the ECRTOOLS tool developed by Prof. Ciucci et al. [S5].

S1.3 Cell Preparation

$S_{3-y}FN_x$ | BZCYYb | $S_{3-y}FN_x$ symmetrical cells were prepared for electrochemical impedance spectroscopy (EIS) measurements. The BZCYYb powder was pressed into a disc and sintered at 1450 °C for 10 h for densification. $S_{3-y}FN_x$ electrode slurry was fabricated by mixing $S_{3-y}FN_x$ powders, isopropanol (AR, ≥99.7%), ethylene glycol (AR, 98%), and glycerol (ACS, ≥99.5%). The electrode slurry was evenly sprayed on the BZCYYb pellet's surface and calcined at 1100 °C for 2 h.

Ni-BZCYYb | BZCYYb | $S_{3-y}FN_x$ single cells were fabricated by a co-pressing method. NiO (standard, FuelCellMaterials. Co.), BZCYYb powder, and starch were thoroughly mixed and pressed as the fuel electrode Ni-BZCYYb, in which the starch functions as the pore former. Subsequently, the pure BZCYYb electrolyte powder was evenly spread and pressed onto the surface of the NiO-BZCYYb, calcined at 1450 °C for 5 h to form a Ni-BZCYYb| BZCYYb half-cell. Afterward, the $S_{3-y}FN_x$ slurry was sprayed on BZCYYb's surface and calcined at 1100 °C for 2 h to produce the single cell.

S1.4 EIS Data Analysis

The Distribution of Relaxation Time (DRT) Method facilitates the extraction of sub-step information about the electrode reaction. The relevant data processing was performed utilizing DRTTOOLS, which was developed by Prof. Ciucci et al. [S6, S7].

DRTTOOLS is specifically designed for EIS data analysis, allowing for the extraction of electrochemical responses at various frequencies. This approach capitalizes on the characteristic time distributions inherent to distinct physicochemical processes. By deconvoluting EIS data into these characteristic distributions based on time scales, the electrode reactions can be effectively identified [S8, S9].

S1.5 Computational Details

Oxygen Reduction Reaction (ORR) proceeds through a four-electron mechanism [S10, S11]：

$$* + O_2 + H^+ + e^- \rightarrow * OOH \qquad (S3)$$

$$* OOH + H^+ + e^- \rightarrow * O + H_2O \qquad (S4)$$

$$* O + H^+ + e^- \rightarrow * OH \qquad (S5)$$

$$* OH + H^+ + e^- \rightarrow H2O \qquad (S6)$$

The Gibbs free energy (ΔG) change for ORR/OER intermediates was determined using the equation (S7) [S12]:

$$\Delta G = \Delta E + \Delta ZPE + T\Delta S - eU \qquad (S7)$$

Here, ΔE represents the energy change in each step, ΔZPE is the change in the zero-point energy calculated from vibrational frequencies, ΔS is the entropy change based on thermodynamics databases, and T denotes the room temperature of 298.15 K. $-eU$ is the standard electrochemical potential of the electron involved in the reaction, obtained when the electrode potential is referenced to the reversible hydrogen electrode. Simultaneously, the standard electrochemical potential of the proton (G_{H_2}) is set to be equivalent to that of a hydrogen atom in gaseous H_2 ($\frac{1}{2}G_{H_2}$). Owing to limitations in characterizing the triplet state of the O_2 molecule within the present DFT framework, the free energy of the O_2 molecule was determined by the following equation [S13]:

$$G_{O_2} = 2G_{H_2O} - 2G_{H_2} + 4.92 \qquad (S8)$$

Corrected free energy values were computed using a plugin for VASP, named VASPKIT.

To quantitatively assess the stability of the perovskite lattice, the formation energy of the Ruddlesden-Popper layered perovskite $S_{3-y}FN_x$ crystal structure was calculated using the formula below [S14, S15]:

$$3SrO + Fe_2O_3 + 0.5O_2 \leftrightarrow Sr_3Fe_2O_7 \qquad (S9)$$

$$3SrO + 0.9Fe_2O_3 + 0.1Nb_2O_5 + 0.4O_2 \leftrightarrow Sr_3Fe_{1.8}Nb_{0.2}O_7 \qquad (S10)$$

$$2.8SrO + 0.9Fe_2O_3 + 0.1Nb_2O_5 + 0.5O_2 \leftrightarrow Sr_{2.8}Fe_{1.8}Nb_{0.2}O_7 \qquad (S11)$$

The formation energy is determined by subtracting the energy of the reactants on the left-hand side from the energy of the products on the right-hand side of the equation. The more negative the formation energy, the higher the stability of the perovskite.

S2 Supplementary Figures

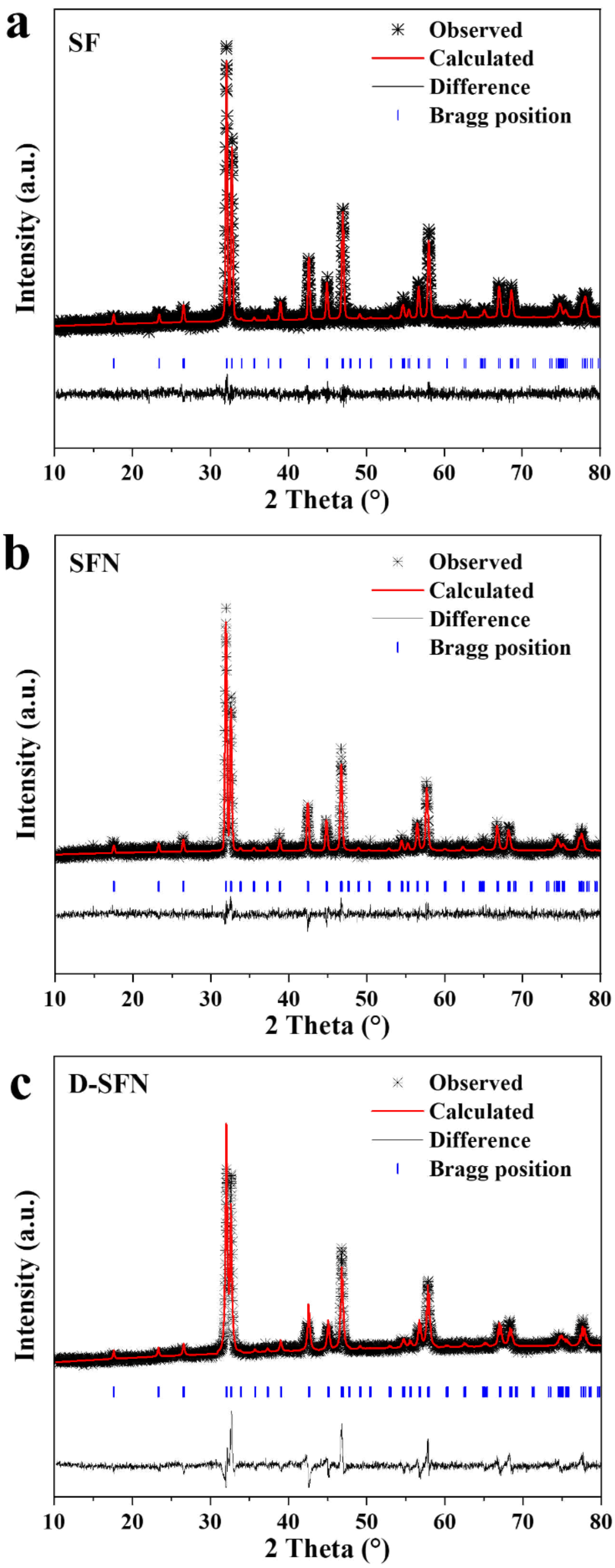

Fig. S1 Refined XRD pattern of $S_{3-y}FN_x$ materials synthesized by sol-gel method

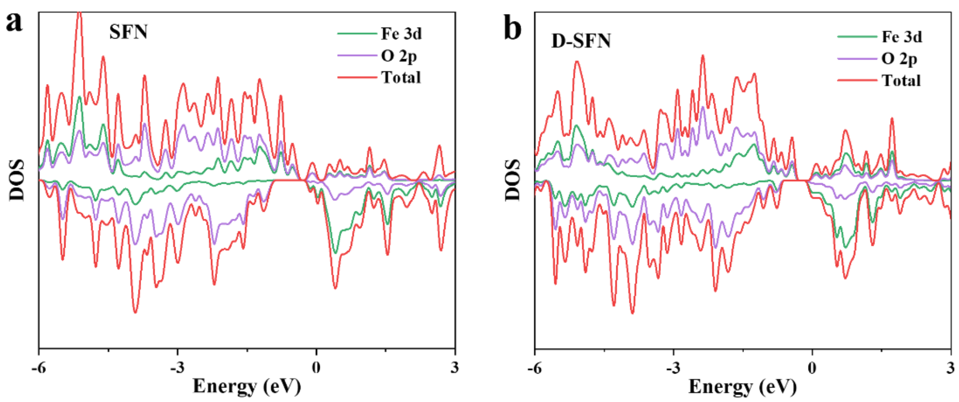

Fig. S2 Density of states (DOS) of SFN and D-SFN

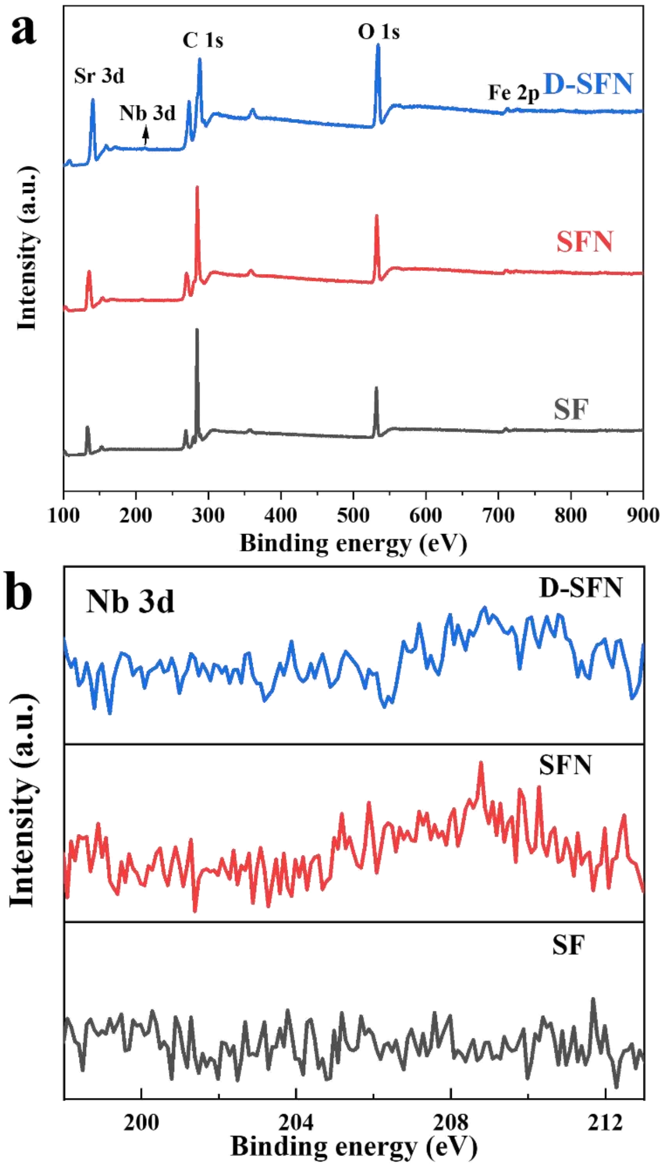

Fig. S3 XPS results of $S_{3-y}FN_x$. **a** XPS detection of the targeted elements. **b** Nb 3d

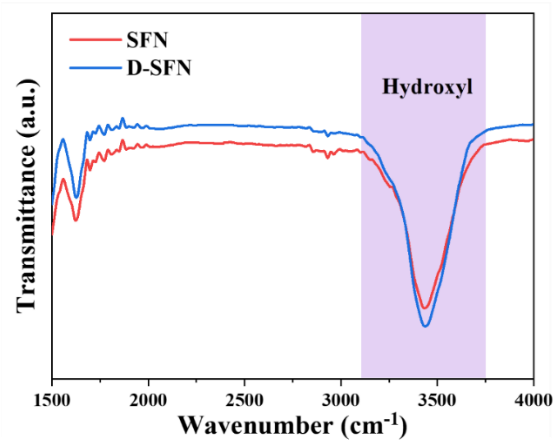

Fig. S4 FT-IR of hydrated SFN and D-SFN powders

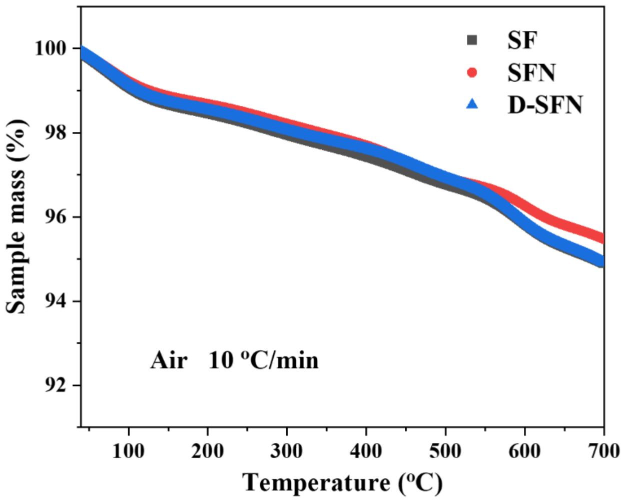

Fig. S5 TG curves of $S_{3-y}FN_x$ samples from room temperature to 700 ºC, with the atmosphere of dry air and the heating rate of 10 ºC/min

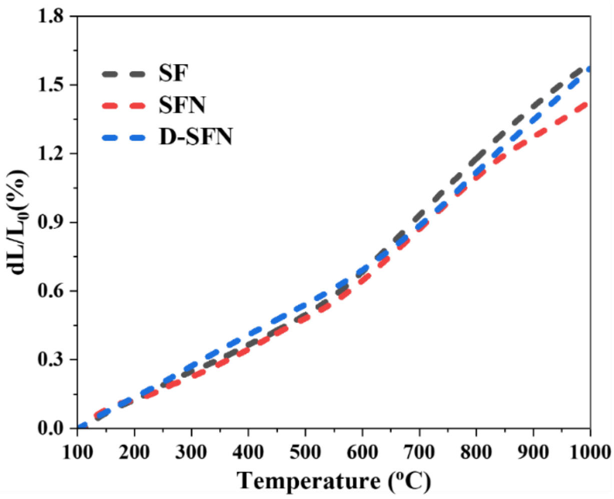

Fig. S6 Thermal expansion coefficient of $S_{3-y}FN_x$ over the temperature range 100-1000 ºC

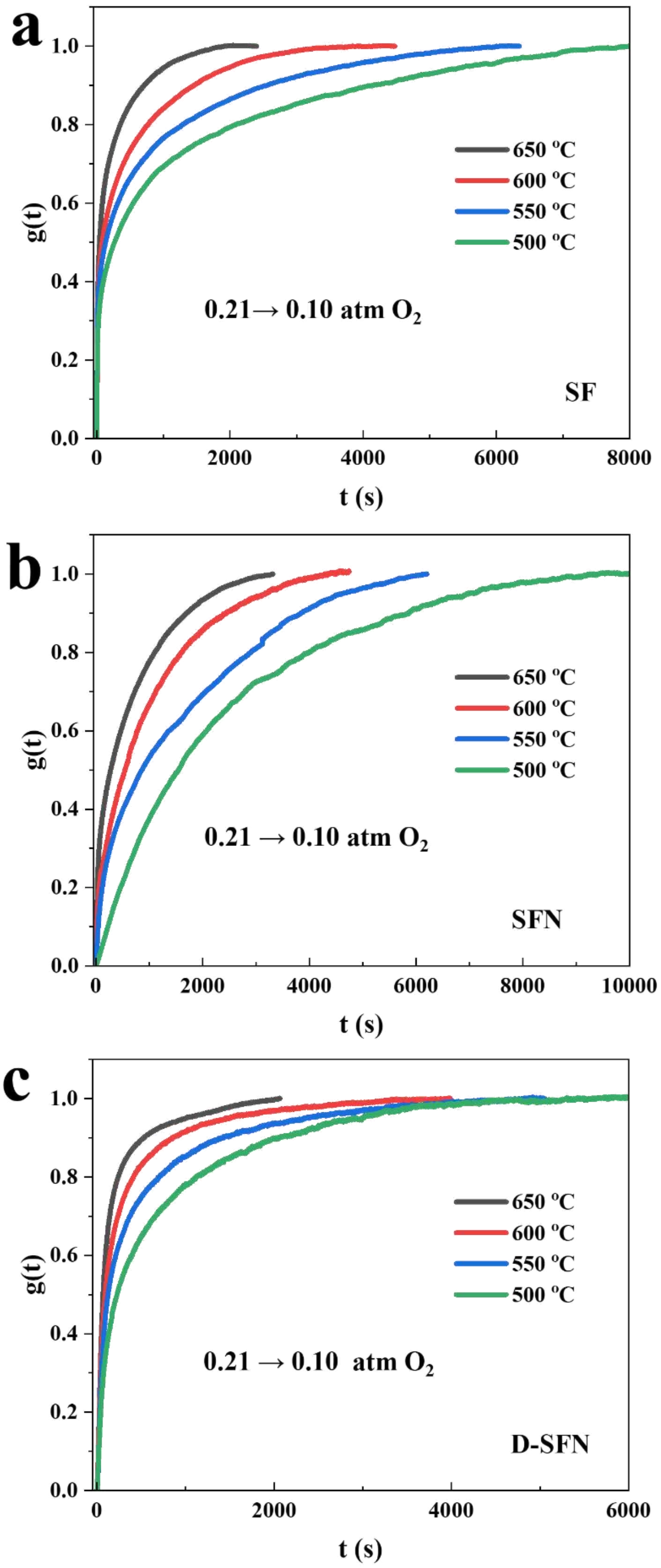

Fig. S7 ECR curve of $S_{3-y}FN_x$ bar samples under changes in oxygen partial pressure

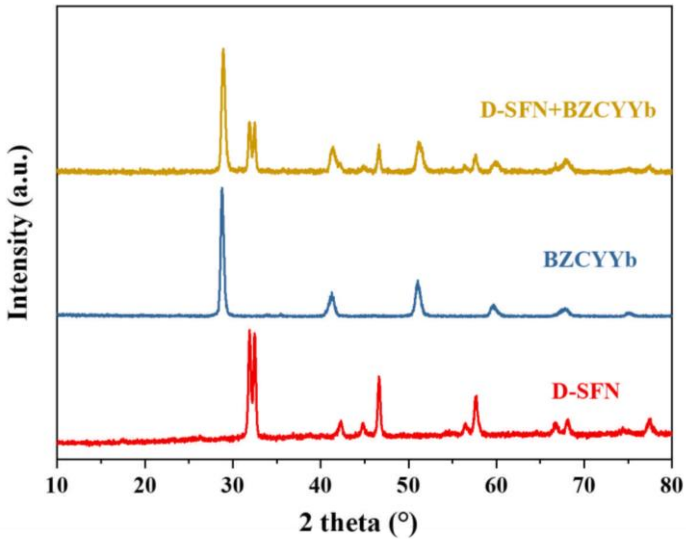

Fig. S8 XRD pattern of BZCYYb powder mixed with D-SFN powder and calcined at 1100 °C for 10 h

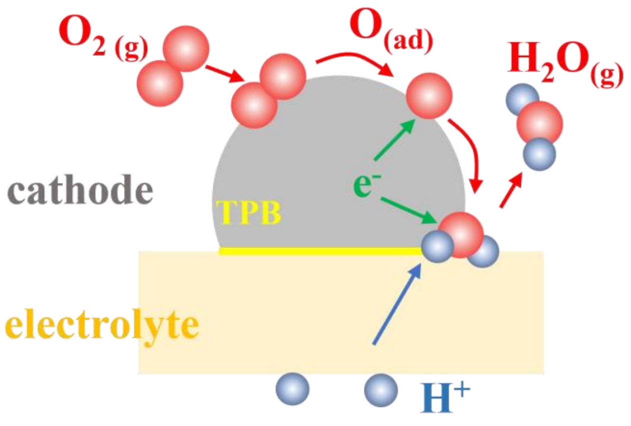

Fig. S9 Schematic diagram of the ORR process of the air electrode in dry air

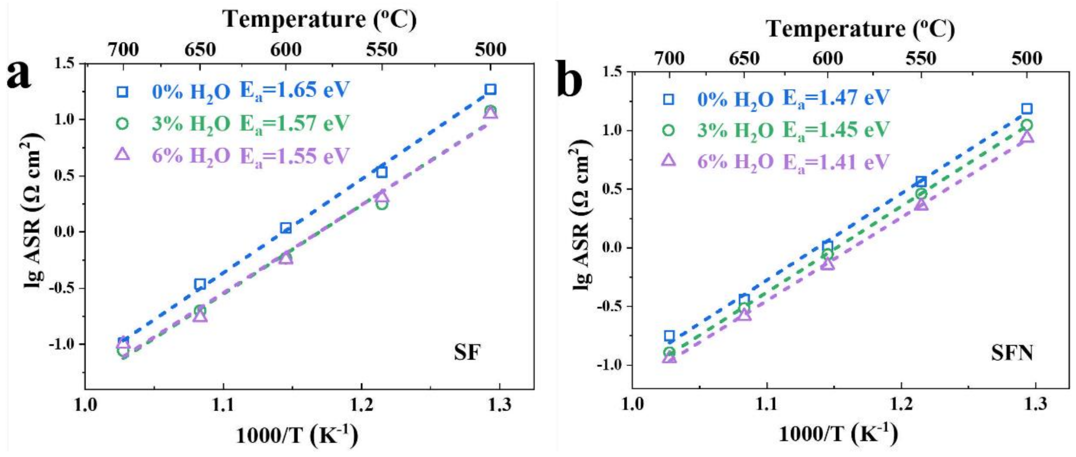

Fig. S10 Arrhenius curves of the symmetrical cells in different steam partial pressure with **a** SF electrode and **b** SFN electrode

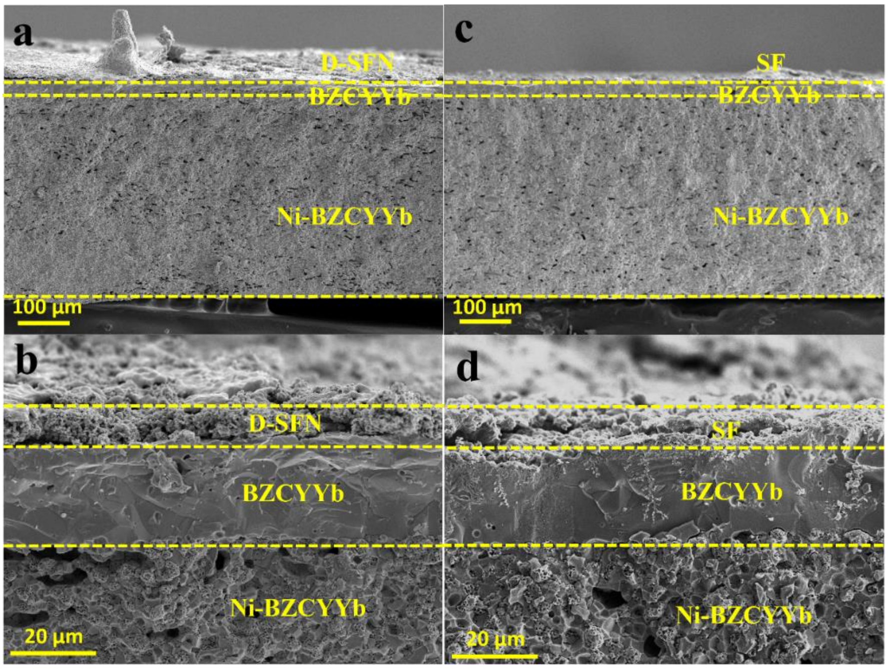

Fig. S11 Cross-section SEM of Ni-BZCYYb|BZCYYb|$S_{3-y}FN_x$ (x=0, 0.2; y=0, 0.2) single cell after test. **a-b** Single cell with D-SFN air electrode. **c-d** Single cell with SF air electrode

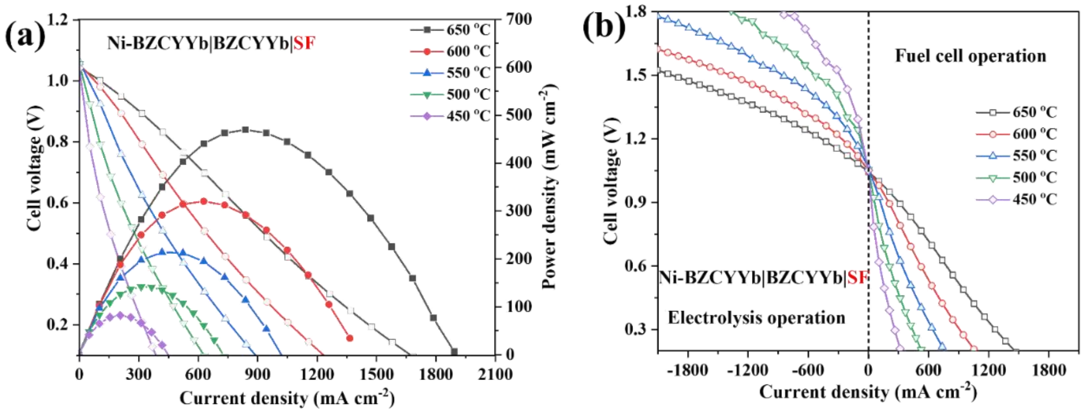

Fig. S12 Electrochemical performance of Ni-BZCYYb|BZCYYb|SF single cell under dry air as fuel and humid air (pH_2O=0.03) as oxidizing atmosphere. **a** Fuel cell power density curve. **b** I-V curves in PCEC and PCFC mode

S3 Supplementary Tables

Table S1 Lattice parameters of $S_{3-y}FN_x$ powders obtained by XRD Rietveld refinement

Samples	a/b (Å)	c (Å)	V (Å^3)	Space group	R_p (%)	R_{wp} (%)	χ^2
SF	3.86634	20.16277	301.405	Im-3m	7.47	9.53	1.10
SFN	3.88744	20.21685	305.521	Im-3m	9.88	12.60	1.16
D-SFN	3.87987	20.10515	302.651	Im-3m	6.09	8.46	3.66

Table S2 Rietveld refinement results derived from the XRD pattern of as-synthesized SF

Element	Wyckoff Symbol	x	y	z	Occupancy
O1	2a	0	0	0	0.058
O2	4e	0	0	0.19329	0.125
Sr1	2b	0	0	0.5	0.062
Sr2	4e	0	0	0.31726	0.125
O3	8g	0	0.5	0.09432	0.250
Fe1	4e	0	0	0.09741	0.125

Table S3 Rietveld refinement results derived from the XRD pattern of as-synthesized SFN

Element	Wyckoff Symbol	x	y	z	Occupancy
O1	2a	0	0	0	0.046
O2	4e	0	0	0.19380	0.125
Sr1	2b	0	0	0.5	0.062
Sr2	4e	0	0	0.31570	0.125
O3	8g	0	0.5	0.09200	0.250
Fe1	4e	0	0	0.09870	0.125

Table S4 Rietveld refinement results derived from the XRD pattern of as-synthesized D-SFN

Element	Wyckoff Symbol	x	y	z	Occupancy
O1	2a	0	0	0	0.046
O2	4e	0	0	0.19380	0.125
Sr1	2b	0	0	0.5	0.062
Sr2	4e	0	0	0.31570	0.125
O3	8g	0	0.5	0.09200	0.250
Fe1	4e	0	0	0.09870	0.125

Table S5 Valence states of Fe and oxygen non-stoichiometry obtained from XPS fitting and iodometric method of $S_{3-y}FN_x$ samples

Samples	XPS fitting		Iodometric method	
	Valence states of Fe	oxygen non-stoichiometry	Valence states of Fe	oxygen non-stoichiometry
SF	+3.52	0.48	+3.49	0.51
SFN	+3.44	0.41	+3.38	0.46
D-SFN	+3.56	0.49	+3.53	0.52

Table S6 D_{chem} and k_{chem} values for SF, SFN, and D-SFN samples at various temperatures

Temperature (°C)	SF		SFN		D-SFN	
	D_{chem} (cm^2 s^{-1})	k_{chem} (cm s^{-1})	D_{chem} (cm^2 s^{-1})	k_{chem} (cm s^{-1})	D_{chem} (cm^2 s^{-1})	k_{chem} (cm s^{-1})
650	2.139E-5	1.671E-4	9.32E-6	8.21E-5	3.755E-5	3.4691E-4
600	1.02E-5	7.956E-5	6.84E-6	6.359E-5	1.968E-5	1.7093E-4
550	5.62E-6	4.298E-5	4.02E-6	3.737E-5	1.085E-5	9.051E-5
500	3.7E-6	2.883E-5	3.09E-6	3.08E-5	7.62E-6	6.539E-5

Table S7 Performance comparison of D-SFN with reported air electrodes in 3% H_2O-air

Air electrode	ASR (Ω cm^2)						Refs.
	750 °C	700 °C	650 °C	600 °C	550 °C	500 °C	
D-SFN		0.047	0.15	0.404	1.209	4.81	This work
$BaCe_{0.4}Sm_{0.2}Fe0.4O_{3-\delta}$ (BCSF)	0.21	0.38	0.78	1.95	5.32		[S16]
$La_{0.6}Sr_{0.4}CoO_{3-\delta}$ (LSC)	0.14	0.55	1.7	3.25	4.00	5.00	[S17]
$Ba_{0.5}Sr_{0.5}Co_{0.8}Fe_{0.2}O_{3-\delta}$ (BSCF)		0.19	0.53	1.60	3.36	9.66	[S18]
$PrBaCo_2O_{5+\delta}$ (PrBC)		0.14	0.29	0.54	1.3	2.78	[S18]
$Pr_2NiO_{4+\delta}$ (PrN)		0.14	0.29	0.58	1.35	3.74	[S18]
$La_{0.6}Sr_{0.4}Fe_{0.8}Co_{0.2}O_{3-\delta}$(LSCF)			1.65	5.12	14.85	64.47	[S19]
$BaCo_{0.4}Fe_{0.4}Zr_{0.2}O_{3-\delta}$ (BCFZ)			0.6	1.03	1.90	3.87	[S19]
$BaCo_{0.4}Fe_{0.4}Zr_{0.15}Y_{0.05}O_3$ (BCFZY0.05)	0.50	1.01	2.10	4.67	12.87	48.63	[S20]
$BaCo_{0.4}Fe_{0.4}Zr_{0.1}Y_{0.1}O_3$ (BCFZY0.1)	0.41	0.89	1.72	4.01	7.69	16.12	[S20]
$BaCo_{0.4}Fe_{0.4}Zr_{0.05}Y_{0.15}O_3$ (BCFZY0.15)	0.67	1.25	2.71	5.43	17.16	50.10	[S20]

Table S8 Performance comparison of reported PCFC under fuel/oxidizing atmosphere of H_2/3% H_2O-air

Air electrode	Electrolyte (Thickness)	Peak power density (mW cm^{-2})						Refs.
		700 °C	650 °C	600 °C	550 °C	500 °C	450 °C	
$BaCe_{0.5}Fe_{0.3}Bi_{0.2}O_{3-\delta}$ (BCFB)	$BaZr_{0.1}Ce_{0.7}Y_{0.2}O_{3-\delta}$ (25 μm)	736	551	362	203			[S21]
$BaFe_{0.9}Bi_{0.1}O_{3-\delta}$ (BFB)	$BaZr_{0.1}Ce_{0.7}Y_{0.2}O_{3-\delta}$ (14 μm)	635	479	338	282			[S22]
$SrSc_{0.175}Nb_{0.025}Co_{0.8}O_{3-\delta}$ (SSNC)	$BaZr_{0.1}Ce_{0.7}Y_{0.2}O_{3-\delta}$ (46 μm)	498	361	262	190	141		[S23]
$LiNi_{0.8}Co_{0.2}O_2$ (LNCO)	$BaZr_{0.1}Ce_{0.7}Y_{0.2}O_{3-\delta}$ (24 μm)		268	221	184			[S24]
$La_{0.7}Sr_{0.3}MnO_{3-\delta}$ (C-LSM73)	BZCY442 (7 μm)	594	434	289	175	98		[S25]
$Sr_{0.9}Ce_{0.1}Fe_{0.8}Ni_{0.2}O_{3-\delta}$ (SCFN)	BZCYYb1711 (26 μm)			420	334	240	135	[S26]
$BaCo_{0.4}Fe_{0.4}Ce_{0.1}Gd_{0.1}O_{3-\delta}$ (BCFCeG)	BZCYYb1711 (30 μm)	663	570	504	395			[S27]
$BaCo_{0.4}Fe_{0.5}Ce_{0.1}$ (BCFCe)	BZCYYb1711 (30 μm)	497	460	406	339			[S27]
$BaCe_{0.4}Fe_{0.4}Co_{0.2}O_{3-\delta}$ (BCFC)	BZCYYb1711 (~70 μm)	335	287	237				[S28]
$La_{1.6}Sr_{0.4}Cu_{0.6}Ni_{0.4}O_{4+\delta}$ (LSCN)	BZCYYb1711 (10 μm)	729	495	272				[S29]
PrNi0.5Co0.5O3-δ(PNC)	BZCYYb4411 (~10 μm)			528	354	230		[S30]
$Ba_{0.5}Sr_{0.5}Co_{0.8}Fe_{0.2}O_{3-\delta}$ (BSCF)	BZCY172 (10 μm)	904	480	289				[S31]
$Ba_{0.4}K_{0.1}Sr_{0.5}Co_{0.8}Fe_{0.2}O_{3-\delta}$ (BKSCF)	BZCY172 (10 μm)	1275	737	441				[S31]
SF	BZCYYb1711 (23 μm)		470	320	214	142	82	This work
D-SFN	BZCYYb1711 (23 μm)		596	483	361	242	165	This work

Table S9 Performance comparison of reported proton-conducting electrolysis cells at an applied voltage of 1.3 V

Air electrode	Fuel/Oxidant	Electrolyte	Current density @1.3 V (A cm^{-2})						Refs.
			700 °C	650 °C	600 °C	550 °C	500 °C	450 °C	
$PrNi_{0.5}Co_{0.5}O_{3-\delta}$ (PNC)	Dry H_2 /10% H_2O-air	BZCYYb (~10 μm)			-0.86	-0.48	-0.34	-0.12	[S30]
$Sr_{0.9}Ce_{0.1}Fe_{0.8}Ni_{0.2}O_{3-\delta}$ (SCFN)	Dry H_2/3% H_2O-air	BZCYYb (26 μm)			-0.36	0.27	0.18	0.09	[S26]
$Pr_2NiO_{4+\delta}$ (PNO)	Dry H_2/40% H_2O-air	$BaZr_{0.2}Ce_{0.6}Y_{0.2}O_{3-\delta}$ (20 μm)		-0.60	-0.34	-0.22			[S32]
$NdBa_{0.5}Sr_{0.5}Co_{1.5}Fe_{0.5}O_{5+\delta}$ (NBSCF-BZCYYb)	90%H_2-10%H_2O/10% H_2O-air	BZCYYb (20 μm)	-2.46	-1.60	-0.73	-0.40			[S33]
$Sr_{2.8}La_{0.2}Fe_2O_{7-\delta}$ (SLF)	3%H_2O-H_2/20% H_2O-air	BZCY (20 μm)	-1.07	-0.72	-0.46				[S34]
$BaCe_{0\cdot6}Zr_{0\cdot3}Y_{0\cdot1}O_{3-\delta}$ (BCZY63)-$BaCo_{0.4}Fe_{0.4}Zr_{0.1}Y_{0-1}O_{3-\delta}$ (BCFZY0.1)	3%H_2O-H_2/12%H_2O-air	$BaCe_{0.7}Zr_{0.1}Y_{0.1}Sm_{0.1}O_{3-\delta}$ (25 μm)			-0.37	-0.22	-0.18	-0.07	[S35]
$La_2NiO_{4+\delta}$ (LN)- $BaCe_{0.5}Zr_{0.3}Dy_{0.2}O_{3-\delta}$ (BCZD)	3%H_2O-H_2/3% H_2O-air	$BaCe_{0.5}Zr_{0.3}Dy_{0.2}O_{3-\delta}$ (30 μm)	-0.30		-0.16				[S36]
$Ba_{0.5}Sr_{0.5}(Co_{0.8}Fe_{0.2})_{0.95}P_{0.05}O_{3-\delta}$ (BSCFP0.05)	H_2/3% H_2O-air	BZCYYb (~10 μm)			-1	-0.62	-0.4		[S37]
$Ba_{0.5}Sr_{0.5}Co_{0.8}Fe_{0.2}O_{3-\delta}$ (BSCF)	H_2/3% H_2O-air	BZCYYb (~10 μm)			-0.64	-0.43	-0.24		[S37]
$BaCo_{0.4}Fe_{0.4}Zr_{0.1}Y_{0.1}O_{3-\delta}$ (BCFZY)	H_2/10% H_2O-air	BZCYYb (~15 μm)			-0.84	-0.58	-0.38	-0.22	[S38]
$Ba_{0.5}Gd_{0.8}La_{0.7}Co_2O_{6-\delta}$-$BaZr_{0.5}Ce_{0.4}Y_{0.1}O_{3-\delta}$ (BGLC587-BZCY541)	20% H_2-80% N_2/30% H_2O-air	BZCY541 (~12 μm)		-1.03	-0.65	-0.38	-0.21		[S39]
$Ba_{0.95}La_{0.05}Fe_{0.8}Zn_{0.2}O_{3-\delta}$ (BLFZ)-BZCYYb	3%H_2O-H_2/10% H_2O-air	BZCYYb (10 μm)	-0.71	-0.46	-0.28				[S40]
$Sr_2Fe_{1.5}Mo_{0.5}O_{6-\delta}$ (SFM)-$BaZrO_3$ (BZY)	H_2/3% H_2O-air	BZY (18 μm)		-0.57	-0.38	-0.20			[S41]
$Nd_{1.95}Ba_{0.05}NiO_{4+\delta}$ (NBN)	3% H_2O-H_2/3% H_2O-air	$BaCe_{0.5}Zr_{0.3}Dy_{0.2}O_{3-\delta}$ (15 μm)	-0.4	-0.28	-0.16	-0.09	-0.09	-0.05	[S42]
SF	H_2/3% H_2O-air	BZCYYb (23 μm)		-0.83	-0.52	-0.29	-0.19	-0.11	This work
D-SFN	H_2/3% H_2O-air	BZCYYb (23 μm)		-1.19	-0.81	-0.57	-0.29	-0.17	This work

Supplementary References

[S1] I. T. Bello, Y. Song, N. Yu, Z. Li, S. Zhao, A. Maradesa, T. Liu, Z. Shao, M. Ni. Evaluation of the electrocatalytic performance of a novel nanocomposite cathode material for ceramic fuel cells. J. Power Sources. **560**, 232722 (2023). https://doi.org/10.1016/j.jpowsour.2023.232722

[S2] I. T. Bello, N. Yu, Y. Song, J. Wang, T.-S. Chan, S. Zhao, Z. Li, Y. Dai, J. Yu, M. Ni. Electrokinetic insights into the triple ionic and electronic conductivity of a novel nanocomposite functional material for protonic ceramic fuel cells. Small. **18**(40), 2203207 (2022). https://doi.org/10.1002/smll.202203207

[S3] Y. Li, K. Gerdes, X. Liu. Oxygen transport kinetics in infiltrated SOFCs cathode by electrical conductivity relaxation technique. J. Electrochem. Soc. **160**(6), F554-F559 (2013). https://doi.org/10.1149/2.056306jes

[S4] J. Ouyang, C.-H. Li, S.-H. Hu. Chemical bulk diffusion coefficient of $Sm_{0.5}Sr_{0.5}CoO_{3-\delta}$ cathode for solid oxide fuel cells. J. Power Sources. **240**, 168-177 (2013). https://doi.org/10.1016/j.jpowsour.2013.03.138

[S5] F. Ciucci. Electrical conductivity relaxation measurements: Statistical investigations using sensitivity analysis, optimal experimental design and ECRTOOLS. Solid State Ionics. **239**, 28-40 (2013). https://doi.org/10.1016/j.ssi.2013.03.020

[S6] T. H. Wan, M. Saccoccio, C. Chen, F. Ciucci. Influence of the discretization methods on the distribution of relaxation times deconvolution: Implementing radial basis functions with DRTtools. Electrochim. Acta. **184**, 483-499 (2015). https://doi.org/10.1016/j.electacta.2015.09.097

[S7] J. Liu, F. Ciucci. The Gaussian process distribution of relaxation times: A machine learning tool for the analysis and prediction of electrochemical impedance spectroscopy data. Electrochim. Acta **331**, 135316 (2020). https://doi.org/10.1016/j.electacta.2019.135316

[S8] F. Ciucci, C. Chen. Analysis of electrochemical impedance spectroscopy data using the distribution of relaxation times: A Bayesian and hierarchical Bayesian approach. Electrochim. Acta. **167**, 439-454 (2015). https://doi.org/10.1016/j.electacta.2015.03.123

[S9] S. J. Cooper, A. Bertei, D. P. Finegan, N. P. Brandon. Simulated impedance of diffusion in porous media. Electrochim. Acta. **251**, 681-689 (2017). https://doi.org/10.1016/j.electacta.2017.07.152

[S10] J. Suntivich, H. A. Gasteiger, N. Yabuuchi, H. Nakanishi, J. B. Goodenough, Y. Shao-Horn. Design principles for oxygen-reduction activity on perovskite oxide catalysts for fuel cells and metal-air batteries. Nat. Chem. **3**(7), 546-550 (2011). https://doi.org/10.1038/nchem.1069

[S11] P. Yao, J. Zhang, Q. Qiu, G. Li, Y. Zhao, F. Yu, Y. Li. Design of a perovskite oxide cathode for a protonic ceramic fuel cell. Ceram. Int. **50**(1, Part B), 2373-2382 (2024). https://doi.org/10.1016/j.ceramint.2023.11.015

[S12] V. Viswanathan, H. A. Hansen, J. Rossmeisl, J. K. Norskov. Unifying the 2e(-) and 4e(-) Reduction of Oxygen on Metal Surfaces. J Phys Chem Lett. **3**(20), 2948-2951 (2012). https://doi.org/10.1021/jz301476w

[S13] M. Bajdich, M. García-Mota, A. Vojvodic, J. K. Nørskov, A. T. Bell. Theoretical investigation of the activity of cobalt oxides for the electrochemical oxidation of water. J. Am. Chem. Soc. **135**(36), 13521-13530 (2013). https://doi.org/10.1021/ja405997s

[S14] C. Chen, F. Ciucci. Designing Fe-based oxygen catalysts by Density Functional Theory calculations. Chem. Mater. **28**(19), 7058-7065 (2016). https://doi.org/10.1021/acs.chemmater.6b02953

[S15] Z. Wang, Y. Wang, J. Wang, Y. Song, M. J. Robson, A. Seong, M. Yang, Z. Zhang, A. Belotti, J. Liu, G. Kim, J. Lim, Z. Shao, F. Ciucci. Rational design of perovskite ferrites as high-performance proton-conducting fuel cell cathodes. Nat. Catal. **5**(9), 777-787 (2022). https://doi.org/10.1038/s41929-022-00829-9

[S16] C. Zhang, H. Zhao. A novel cobalt-free cathode material for proton-conducting solid oxide fuel cells. J. Mater. Chem. **22**(35), 18387-18394 (2012). https://doi.org/10.1039/C2JM32627B

[S17] A. A. Samat, A. A. Jais, M. R. Somalu, N. Osman, A. Muchtar, K. L. Lim. Electrical and electrochemical characteristics of $La_{0.6}Sr_{0.4}CoO_{3-\delta}$ cathode materials synthesized by a modified citrate-EDTA sol-gel method assisted with activated carbon for proton-conducting solid oxide fuel cell application. J. Sol-Gel Sci. Technol. **86**(3), 617-630 (2018). https://doi.org/10.1007/s10971-018-4675-1

[S18] A. Grimaud, F. Mauvy, J. M. Bassat, S. Fourcade, L. Rocheron, M. Marrony, J. C. Grenier. Hydration properties and rate determining Steps of the oxygen reduction reaction of perovskite-related oxides as H^+-SOFC cathodes. J. Electrochem. Soc. **159**(6), B683 (2012). https://doi.org/10.1149/2.101205jes

[S19] M. Shang, J. Tong, R. O'Hayre. A promising cathode for intermediate temperature protonic ceramic fuel cells: $BaCo_{0.4}Fe_{0.4}Zr_{0.2}O_{3-\delta}$. Rsc. Adv. **3**(36), 15769-15775 (2013). https://doi.org/10.1039/C3RA41828F

[S20] C. Duan, J. Tong, M. Shang, S. Nikodemski, M. Sanders, S. Ricote, A. Almansoori, R. O’Hayre. Readily processed protonic ceramic fuel cells with high performance at low temperatures. Science **349**(6254), 1321-1326 (2015). https://doi.org/10.1126/science.aab3987

[S21] D. Shan, Z. Gong, Y. Wu, L. Miao, K. Dong, W. Liu. A novel $BaCe_{0.5}Fe_{0.3}Bi_{0.2}O_{3-\delta}$ perovskite-type cathode for proton-conducting solid oxide fuel cells. Ceram. Int.

43(4), 3660-3663 (2017). https://doi.org/10.1016/j.ceramint.2016.11.206

[S22] Y. Xia, X. Xu, Y. Teng, H. Lv, Z. Jin, D. Wang, R. Peng, W. Liu. A novel $BaFe_{0.8}Zn_{0.1}Bi_{0.1}O_{3-\delta}$ cathode for proton conducting solid oxide fuel cells. Ceram. Int. **46**(16, Part A), 25453-25459 (2020). https://doi.org/10.1016/j.ceramint.2020.07.015

[S23] A. Zhu, G. Zhang, T. Wan, T. Shi, H. Wang, M. Wu, C. Wang, S. Huang, Y. Guo, H. Yu, Z. Shao. Evaluation of $SrSc_{0.175}Nb_{0.025}Co_{0.8}O_{3-\delta}$ perovskite as a cathode for proton-conducting solid oxide fuel cells: The possibility of in situ creating protonic conductivity and electrochemical performance. Electrochim. Acta. **259**, 559-565 (2018). https://doi.org/10.1016/j.electacta.2017.11.037

[S24] L. Fan, P.-C. Su. Layer-structured $LiNi_{0.8}Co_{0.2}O_2$: A new triple ($H^+/O^{2-}/e^-$) conducting cathode for low temperature proton conducting solid oxide fuel cells. J. Power Sources. **306**, 369-377 (2016). https://doi.org/10.1016/j.jpowsour.2015.12.015

[S25] N. Wang, S. Hinokuma, T. Ina, C. Zhu, H. Habazaki, Y. Aoki. Mixed proton-electron-oxide ion triple conducting manganite as an efficient cobalt-free cathode for protonic ceramic fuel cells. J. Mater. Chem. A. **8**(21), 11043-11055 (2020). https://doi.org/10.1039/D0TA03899G

[S26] Y. Song, J. Liu, Y. Wang, D. Guan, A. Seong, M. Liang, M. J. Robson, X. Xiong, Z. Zhang, G. Kim, Z. Shao, F. Ciucci. Nanocomposites: A new opportunity for developing highly active and durable bifunctional air electrodes for reversible protonic ceramic cells. Adv. Energy Mater. **11**(36), 2101899 (2021). https://doi.org/10.1002/aenm.202101899

[S27] L. Zhang, S. Yang, S. Zhang, Y. Yang. Cerium and Gadolinium co-doped perovskite oxide for a protonic ceramic fuel cell cathode. Int. J. Hydrogen Energy. **44**(51), 27921-27929 (2019). https://doi.org/10.1016/j.ijhydene.2019.09.057

[S28] Z. Zhao, J. Cui, M. Zou, S. Mu, H. Huang, Y. Meng, K. He, K. S. Brinkman, J. Tong. Novel twin-perovskite nanocomposite of Ba-Ce-Fe-Co-O as a promising triple conducting cathode material for protonic ceramic fuel cells. J. Power Sources. **450**, 227609 (2020). https://doi.org/10.1016/j.jpowsour.2019.227609

[S29] J. Ma, Y. Pan, Y. Wang, Y. Chen. A Sr and Ni doped Ruddlesden−Popper perovskite oxide $La_{1.6}Sr_{0.4}Cu_{0.6}Ni_{0.4}O_{4+\delta}$ as a promising cathode for protonic ceramic fuel cells. J. Power Sources. **509**, 230369 (2021). https://doi.org/10.1016/j.jpowsour.2021.230369

[S30] H. Ding, W. Wu, C. Jiang, Y. Ding, W. Bian, B. Hu, P. Singh, C. J. Orme, L. Wang, Y. Zhang. Self-sustainable protonic ceramic electrochemical cells using a triple conducting electrode for hydrogen and power production. Nat. Commun. **11**(1), 1907 (2020). https://doi.org/10.1038/s41467-020-15677-z

[S31] X. Xu, H. Wang, M. Fronzi, X. Wang, L. Bi, E. Traversa. Tailoring cations in a

perovskite cathode for proton-conducting solid oxide fuel cells with high performance. J. Mater. Chem. A. **7**(36), 20624-20632 (2019). https://doi.org/10.1039/C9TA05300J

[S32] W. Li, B. Guan, L. Ma, S. Hu, N. Zhang, X. Liu. High performing triple-conductive $Pr_2NiO_{4+\delta}$ anode for proton-conducting steam solid oxide electrolysis cell. J. Mater. Chem. A. **6**(37), 18057-18066 (2018). https://doi.org/10.1039/C8TA04018D

[S33] J. Kim, A. Jun, O. Gwon, S. Yoo, M. Liu, J. Shin, T.-H. Lim, G. Kim. Hybrid-solid oxide electrolysis cell: A new strategy for efficient hydrogen production. Nano Energy. **44**, 121-126 (2018). https://doi.org/10.1016/j.nanoen.2017.11.074

[S34] D. Huan, W. Wang, Y. Xie, N. Shi, Y. Wan, C. Xia, R. Peng, Y. Lu. Investigation of real polarization resistance for electrode performance in proton-conducting electrolysis cells. J. Mater. Chem. A. **6**(38), 18508-18517 (2018). https://doi.org/10.1039/C8TA06862C

[S35] Y. Meng, J. Gao, H. Huang, M. Zou, J. Duffy, J. Tong, K. S. Brinkman. A high-performance reversible protonic ceramic electrochemical cell based on a novel Sm-doped $BaCe_{0.7}Zr_{0.1}Y_{0.2}O_{3-\delta}$ electrolyte. J. Power Sources. **439**, 227093 (2019). https://doi.org/10.1016/j.jpowsour.2019.227093

[S36] J. Lyagaeva, N. Danilov, G. Vdovin, J. Bu, D. Medvedev, A. Demin, P. Tsiakaras. A new Dy-doped $BaCeO_3$–$BaZrO_3$ proton-conducting material as a promising electrolyte for reversible solid oxide fuel cells. J. Mater. Chem. A. **4**(40), 15390-15399 (2016). https://doi.org/10.1039/C6TA06414K

[S37] Z. Liu, D. Cheng, Y. Zhu, M. Liang, M. Yang, G. Yang, R. Ran, W. Wang, W. Zhou, Z. Shao. Robust bifunctional phosphorus-doped perovskite oxygen electrode for reversible proton ceramic electrochemical cells. Chem. Eng. J. **450**, 137787 (2022). https://doi.org/10.1016/j.cej.2022.137787

[S38] M. Liang, Y. Song, D. Liu, L. Xu, M. Xu, G. Yang, W. Wang, W. Zhou, R. Ran, Z. Shao. Magnesium tuned triple conductivity and bifunctionality of $BaCo_{0.4}Fe_{0.4}Zr_{0.1}Y_{0.1}O_{3-\delta}$ perovskite towards reversible protonic ceramic electrochemical cells. Appl. Catal. B-Environ. **318**, 121868 (2022). https://doi.org/10.1016/j.apcatb.2022.121868

[S39] H. Zheng, M. Riegraf, N. Sata, R. Costa. A double perovskite oxygen electrode in Zr-rich proton conducting ceramic cells for efficient electricity generation and hydrogen production. J. Mater. Chem. A. **11**(20), 10955-10970 (2023). https://doi.org/10.1039/D3TA01298K

[S40] J. Jing, Z. Lei, Z. Zheng, H. Wang, P. Zhang, Z. Wang, H. Xu, Z. Yang. Triple conducting perovskite $Ba_{0.95}La_{0.05}Fe_{0.8}Zn_{0.2}O_{3-\delta}$ as oxygen electrode for reversible protonic ceramic cells. Int. J. Hydrogen Energy. **48**(24), 9037-9045 (2023). https://doi.org/10.1016/j.ijhydene.2022.12.019

[S41] L. Lei, J. Zhang, R. Guan, J. Liu, F. Chen, Z. Tao. Energy storage and hydrogen production by proton conducting solid oxide electrolysis cells with a novel heterogeneous design. Energ. Convers. Manage. **218**, 113044 (2020). https://doi.org/10.1016/j.enconman.2020.113044

[S42] N. Danilov, J. Lyagaeva, G. Vdovin, E. Pikalova, D. Medvedev. Electricity/hydrogen conversion by the means of a protonic ceramic electrolysis cell with $Nd_2NiO_{4+\delta}$-based oxygen electrode. Energ. Convers. Manage. **172**, 129-137 (2018). https://doi.org/10.1016/j.enconman.2018.07.014
